# Continued permafrost ecosystem carbon loss under net-zero and negative emissions

**DOI:** 10.1126/sciadv.adn8819

**Published:** 2025-02-12

**Authors:** So-Won Park, Jin-Hyuk Mun, Hanna Lee, Norman J. Steinert, Soon-Il An, Jongsoo Shin, Jong-Seong Kug

**Affiliations:** ^1^ School of Earth and Environmental Sciences, Seoul National University, Seoul, South Korea.; ^2^ Department of Biology, Norwegian University of Science and Technology, Trondheim, Norway.; ^3^ NORCE Climate and Environment, Bjerknes Centre for Climate Research, Bergen, Norway.; ^4^ CICERO Center for International Climate Research, Oslo, Norway.; ^5^ Department of Atmospheric Sciences/Irreversible Climate Change Research Center, Yonsei University, Seoul, South Korea.; ^6^ Woods Hole Oceanographic Institution, Woods Hole, MA, USA.

## Abstract

The loss of ecosystem carbon (the sum of vegetation, litter, and soil carbon) may occur in a permafrost region under mitigation pathways, which could reduce the efficiency of carbon dioxide removal. Here, we investigate changes in permafrost under net-zero and negative emissions, based on idealized emission-driven simulations using a state-of-the-art Earth system model. While acting as a net ecosystem carbon sink during most of the positive emission phase, permafrost becomes a net ecosystem carbon source just before reaching net-zero and negative emissions. Permafrost slowly recovers, especially in regions with high organic carbon content, and net ecosystem carbon loss persists until the end of simulations, resulting in a cumulative net ecosystem carbon loss of approximately 14 petagrams of carbon (PgC) in both scenarios. In addition, methane emissions increase under net-zero and negative emissions, due to the irreversibility of the inundated areas. We conclude that the permafrost ecosystem carbon loss may continue under net-zero and negative emissions, which could hinder climate change mitigation efforts.

## Teaser

Permafrost ecosystem carbon loss may continue under net-zero and negative emissions, hindering climate mitigation efforts.

## INTRODUCTION

Anthropogenic carbon dioxide (CO_2_) emissions have been causing long-term global warming with adverse effects on nature and people, which will continue to cause further changes in the climate system, ecosystem, and humanity (*1*–*4*). To reduce climate-related risks, the 2015 Paris Agreement aims to pursue efforts to limit global warming to well below 2°C and preferably to 1.5°C, compared to the preindustrial level (*5*). Most analyzed pathways, consistent with these climate targets, involve reaching net-zero emissions and transitioning to net-negative emissions through the use of carbon dioxide removal (CDR) techniques (*6*–*9*). In assessing the effectiveness and potential risks of mitigation pathways that rely on CDR, the reversibility, hysteresis, or tipping points of the Earth’s system are key factors to be considered (*10*). It has been reported that many aspects of the physical climate system, such as temperature, precipitation, and sea ice, are largely reversible on the global scale, although in general with some hysteresis (dependence of the state of a system on its history) (*11*–*16*). However, among several components of the Earth system, carbon (C) release from permafrost is expected to be irreversible, manifesting fully on longer timescales (*11*, *17*).

Permafrost-affected soils in the northern circumpolar permafrost region store approximately 1460 to 1600 PgC (*18*, *19*). As the Earth warms, permafrost thaw will lead to the decay of organic carbon in permafrost-affected soils, releasing additional greenhouse gases such as CO_2_ and methane (CH_4_) into the atmosphere (*19*–*24*). This carbon release from the permafrost-affected soils has the potential to further amplify human-induced global warming (*22*, *25*, *26*). In addition, as the climate warms, plants are expected to grow faster and absorb more carbon from the atmosphere, which could help offset some of the carbon losses from the soil (*27*, *28*). Because of these opposing effects, the changes in permafrost net carbon balance in response to net-zero and negative emissions are expected to be complex. This has a critical impact on the future responses of the coupled carbon-climate system to anthropogenic emissions. Therefore, we need to understand the changes in a permafrost region under mitigation pathways: When and how much ecosystem carbon (sum of vegetation, litter, and soil C) will be emitted from the permafrost region and what is the ratio of CO_2_ to CH_4_?

To date, the reversibility and hysteresis of permafrost extent and permafrost C cycle have been investigated on the basis of idealized CO_2_ ramp-up and ramp-down forcing experiments using Earth system models (ESMs) (*11*, *29*). The permafrost area exhibits hysteresis as a function of CO_2_ concentration and global mean surface temperature, with less permafrost extent in the CO_2_ ramp-down period due to its slow recovery (*11*, *30*). It is also suggested that although the permafrost extent would eventually be reversible, the loss of organic carbon in permafrost while thawed would be irreversible (*11*, *29*, *31*). However, the carbon-climate feedback is not fully considered in previous studies because they are based on idealized concentration-driven simulations (*32*). In addition, the inferred emissions pathway is highly discontinuous and impractical (*10*, *33*). Therefore, it is still unclear how changes in permafrost and its carbon dynamics will affect the climate system under mitigation pathways.

Recent studies based on various emission-driven simulations involving net-zero and negative emissions phases have shown that changes in permafrost extent depend on temperature, which is influenced not only by radiative forcing but also by the behavior of the Atlantic Meridional Overturning Circulation (AMOC) (*10*, *34*). During the positive emission phase, permafrost degrades due to global warming, and then after the onset of net-zero and negative emissions, permafrost extent increases again with decreasing temperature (*10*, *34*). However, as the weakened AMOC begins to recover, surface warming occurs in the northern mid-to-high latitudes, which leads to the permafrost rethaw, although its timing and magnitude vary between scenarios and models. These pronounced temperature fluctuations in the northern high latitudes under net-zero and negative emissions would have ramifications on both Arctic ecosystems and permafrost soils, complicating the understanding of the net ecosystem C balance in permafrost regions.

Here, we aim to understand changes in permafrost depending on CDR options (net-zero or negative emissions) and its role in climate change. Accordingly, this study addresses the following questions: (i) How will permafrost extent and active layer thickness (ALT) change under net-zero and negative emissions? (ii) How much permafrost ecosystem carbon will be emitted or absorbed along the mitigation emission pathways? (iii) What are the major processes responsible for these changes? (iv) Does hysteresis of permafrost exist under net-zero and negative emissions, and if so, how will it affect the net ecosystem carbon balance?

**Fig. 1.**
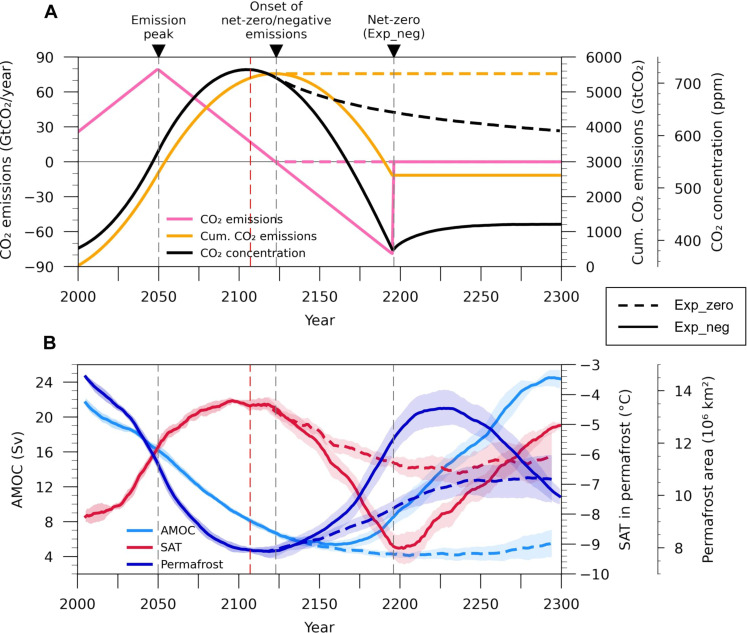
Evolution of the climate system and permafrost area in idealized net-zero and negative emissions scenarios. (**A**) Time series of annual anthropogenic CO_2_ emissions, cumulative CO_2_ emissions, and annual mean atmospheric CO_2_ concentration. (**B**) Time series of the 11-year running mean of AMOC strength, permafrost area, and annual mean SAT. The AMOC strength is defined as the average of the annual mean Atlantic meridional ocean stream function from 35° to 45°N at a depth of 1000 m. The SAT is averaged over the initial permafrost domain. The solid and dashed lines show the ensemble mean values for Exp_neg and Exp_zero, respectively. The shading indicates the 95% confidence interval based on the bootstrap method. The peak of anthropogenic emissions and the onset of negative emissions are indicated by the gray dashed vertical line. The point of maximum CO_2_ levels is indicated by the red dashed vertical line.

To address these questions, we performed emission-driven simulations using a state-of-the-art ESM that includes a representation of permafrost dynamics [Community Earth System Model version 2 (CESM2) coupled with the Community Land Model version 5 (CLM5); see Materials and Methods] (*35*). The simulations are based on two idealized and continuous emission pathways involving net-zero or negative emissions (Exp_zero or Exp_neg) after the positive anthropogenic CO_2_ emissions (Fig. 1A; see Materials and Methods). Here, near-surface permafrost is defined as areas where the simulated maximum ALT is shallower than 4 m (*19*, *34*, *36*). The temperature and carbon stock in the permafrost region are calculated on the basis of the initial permafrost domain (fig. S1).

## RESULTS

### Changes in near-surface permafrost under net-zero and negative emission pathways

The near-surface air temperature (SAT) in the permafrost region almost follows the changes in atmospheric CO_2_ concentrations (Fig. 1). In addition, SAT is substantially affected by the behavior of the AMOC, mainly later in the experiments. SAT increases by ~4 K during the positive emissions phase and then decreases with decreasing CO_2_ concentrations after the onset of net-zero and negative emissions. In Exp_zero, atmospheric CO_2_ concentration declines gradually from its peak to the end of the simulation [730 to 610 parts per million (ppm)] but remains higher than its initial level, keeping permafrost regions warmer even beyond the peak of CO_2_ levels. Despite declining CO_2_ concentrations, the SAT rises slightly after year 2250 due to the partial recovery of the AMOC. These results are consistent with previous results shown in the shared socioeconomic pathway (SSP) SSP5-3.4-overshoot (SSP5-3.4) overshoot scenario and an idealized CO_2_ ramp-up and ramp-down experiment (*10*, *31*, *34*, *37*, *38*).

In Exp_neg, from the onset of the negative emissions phase, the CO_2_ concentration gradually declines, results in surface cooling, and eventually reaches its initial state. At the time of the initial recovery of CO_2_, the temperature is about 1°C lower than its initial state due to the cooling caused by the weakening of the AMOC. Similarly, in Exp_zero, the SAT recovers faster than the rate of change of CO_2_ due to the cooling induced by the AMOC. During the restoring period (phase of net-zero emissions) in Exp_neg, the AMOC exhibits a strong recovery unlike in Exp_zero, and thus, the temperature in the Northern Hemisphere increases considerably. As a result, considerable warming occurs in the permafrost region, comparable to its state at the CO_2_ peak, although CO_2_ concentrations increase slightly (~50 ppm) compared to the initial state. That is, if negative emission is deployed after the phase of positive emissions, then a strong temperature fluctuation would occur in the permafrost regions, which is consistent with previous studies (*10*, *34*).

The extent of near-surface permafrost highly depends on the SAT, consistent with previous studies (*11*, *30*, *34*, *39*). In Exp_zero, a thawing-refreezing pattern emerges according to the warming-cooling cycle. In Exp_neg, the temperature fluctuations (warming-cooling-warming) lead to a thawing-refreezing-rethawing cycle (Fig. 1B and fig. S1). In Exp_zero, during the early phase of net-zero emissions (from year 2123 to 2197), the temperature recovers by ~49%, whereas the permafrost extent only recovers by 24%. In Exp_neg, it cannot fully recover despite the temperature in year 2197 being lower than the initial state, suggesting the presence of hysteresis in permafrost extent. Particularly along the initial boundary of permafrost zone in North America, the near-surface permafrost does not recover, resulting in a slow recovery of permafrost extent (fig. S1).

### Evolution of net ecosystem C balance in permafrost under mitigation pathways

**Fig. 2.**
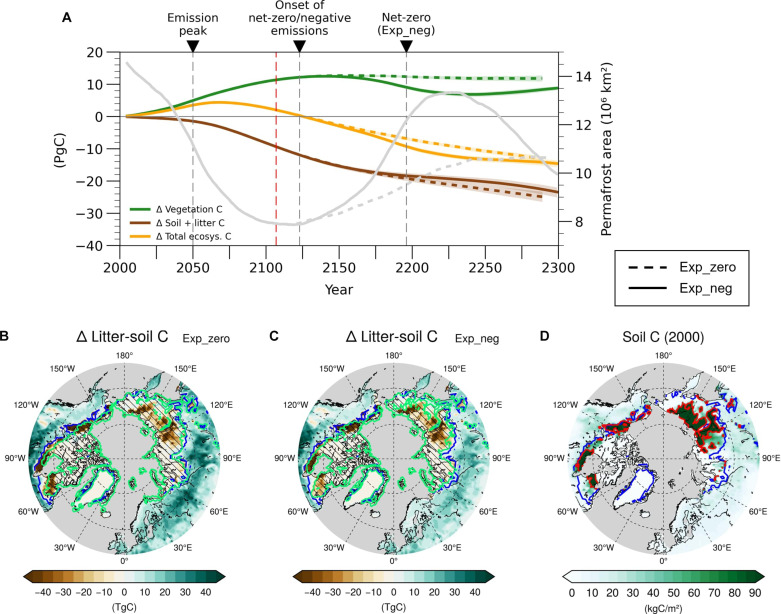
Changes in terrestrial carbon stocks in the permafrost region. (**A**) Time series of the 11-year running mean of annual mean vegetation, litter-soil, and total ecosystem carbon pool anomalies relative to the year 2000. All values are averaged over the initial permafrost domain. Gray solid lines show the evolution of the permafrost area. (**B**) Litter-soil carbon pool anomaly in the year 2197 relative to the year 2000 in Exp_zero and (**C**) Exp_neg. The simulated permafrost boundaries for the years of 2000 and 2197 are shown as blue and green lines, respectively. Hatched areas indicate the permafrost extent for each period. (**D**) Initial soil carbon content in the northern mid-high latitudes. Regions with high organic content are defined by an organic matter content of >75 kgC/m^2^, and their boundary is shown as a red line. All calculations were conducted after taking the 11-year running mean and then averaging (ensemble mean).

Next, we examined the temporal evolution of the ecosystem carbon cycle in the permafrost region (Fig. 2A). During the positive emissions phase, a consistent trend emerges with vegetation C pool steadily increasing, possibly due to the CO_2_ fertilization effect and pan-Arctic warming (*27*), while the sum of litter and soil C pool decreases. In the first half, vegetation C uptake exceeds the loss of litter and soil C through respiration, thereby increasing the total ecosystem C (fig. S2). However, from around year 2060, litter-soil C begins to decline sharply and thus overwhelms the increase in vegetation C, resulting in a net loss of ecosystem C in the permafrost region. As a result, the total ecosystem C anomaly returns to zero at the beginning of the net-zero and negative emissions.

After the cessation of the positive emissions in Exp_zero, net C uptake by vegetation, i.e., net primary production (NPP), begins to decrease as CO_2_ concentration and temperature decline (Fig. 1 and fig. S2), but vegetation C remains stable, possibly due to the inherent lag in the terrestrial C pool (*40*, *41*). However, because of the continuous loss of litter and soil C pool caused by high temperature, the total ecosystem C gradually decreases, thereby resulting in a total ecosystem C loss of ~14 PgC by the end of the simulation (Fig. 2A).

In Exp_neg, during the negative emission phase (from year 2124 to 2196), as CO_2_ and SAT recover, NPP decreases and returns close to its initial state, which leads to the decline in vegetation carbon of ~3.5 PgC (Figs. 1 and 2A and fig. S2). In addition, during the same period, the respiratory C loss continues due to an incomplete recovery of permafrost extent, resulting in a reduction in litter-soil C of ~6.2 PgC (Figs. 1B and 2 and fig. S1). This reduction can also be partially attributed to the delayed increase in respiration resulting from the previously increased NPP (*29*, *41*). Although the permafrost area recovers more than twice as much in Exp_neg in the same time period, litter-soil C emissions are similar in the two scenarios. This is because the intensive respiratory C loss occurs prominently along the initial boundary of permafrost in North America, which exhibits slow recovery due to its high soil organic matter (SOM) content (Fig. 2, B to D, and figs. S1 and S3).

In Exp_neg, during the restoring period, as the temperature increases due to the recovery of AMOC and CO_2_ concentrations rebound, vegetation C slightly increases again. However, as temperature rises and permafrost degrades, litter and soil C continue to decrease until the end of the simulation. As a result, despite the deployment of artificial CO_2_ removal of ~800 PgC only in Exp_neg, the magnitude of change in total ecosystem C by 2300 is similar between the two scenarios. In summary, during the positive emission period, permafrost acts as a net C sink but thereafter becomes a net C source. The ecosystem C loss in permafrost regions under net-zero and negative emissions can hinder our efforts in climate change mitigation.

### Irreversible changes in CH_4_ emissions under net-zero and negative emissions

The temporal evolution of CH_4_ production, oxidation, and CH_4_ emission rates is quite similar (fig. S4). As the total production of CH_4_ increased, the amount of oxidation of CH_4_ to CO_2_ and CH_4_ released into the atmosphere would have increased. In CLM5, methane production is related to the estimate of heterotrophic respiration corrected by multiple factors associated with the inundated fraction, soil temperature, pH, and redox potential (*42*, *43*). In particular, changes in soil moisture are known to have a critical impact on CH_4_ production. Soil drying decreases CH_4_ emissions through a reduction of the inundated fraction and a deepening of the water table that reduces anoxia in the non-inundated fraction (*44*). On the other hand, larger inundated fractions and a shallow water table lead to an increase in CH_4_ emissions due to the use of an anaerobic decomposition pathway, but total C loss may decrease as a result of reduced heterotrophic respiration due to oxygen limitation (*45*–*47*).

**Fig. 3.**
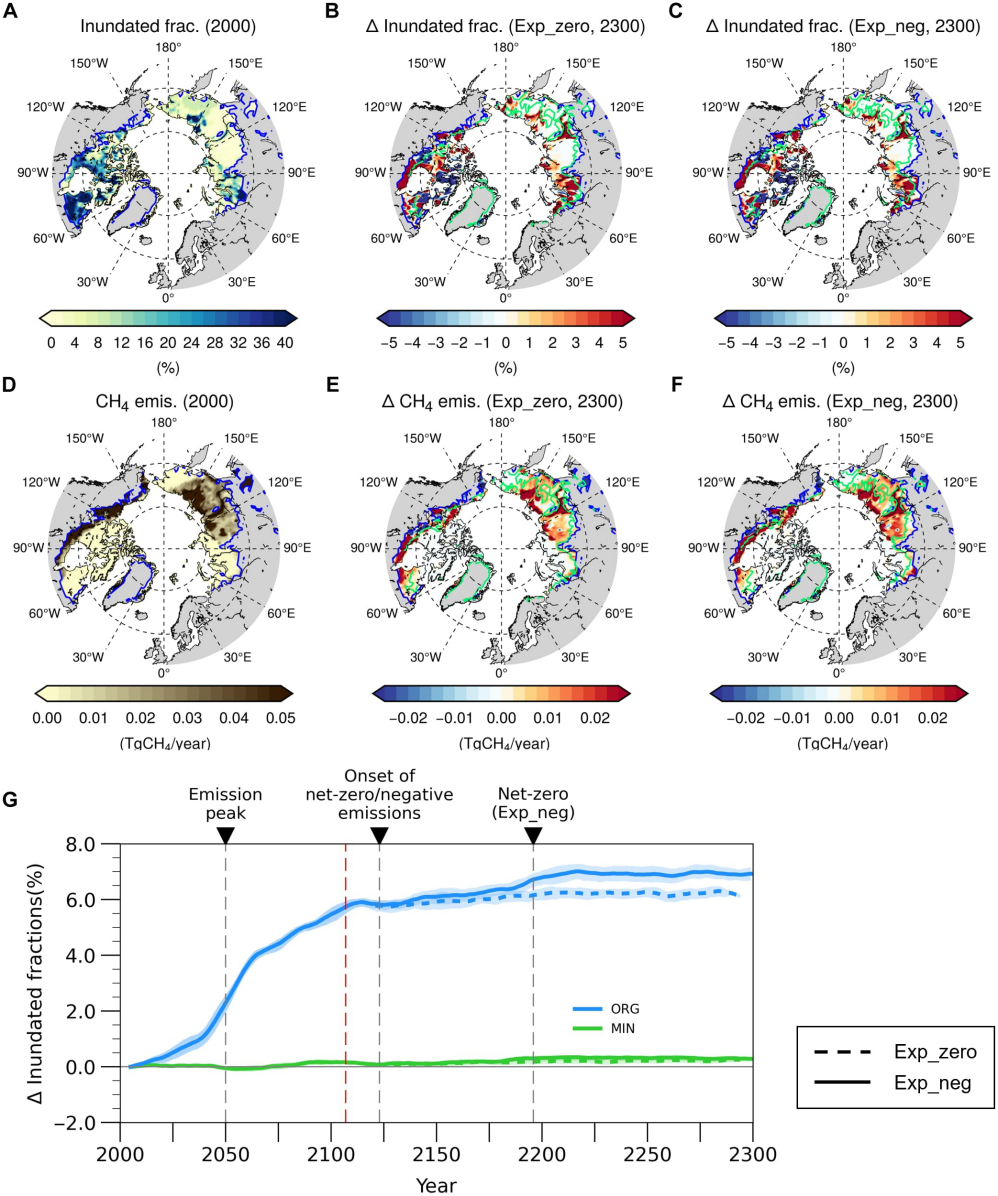
Regional differences in inundated fractions and CH_4_ emissions. (**A**) Initial state of the annual mean inundated fractions and (**D**) CH_4_ emissions in the permafrost region. (**B** and **C**) Changes in annual mean inundated fractions and (**E** and **F**) CH_4_ emissions in the year 2300 relative to year 2000 (B and E) in Exp_zero and (C and F) Exp_neg. (**G**) Time series of the 11-year running mean of annual mean inundated fractions, averaged over the initial permafrost domain with predominantly xorganic and mineral soils. Regions with high organic content (ORG) are defined by an organic matter content of >75 kgC/m^2^, and regions with relatively low organic matter content (MIN) are defined by an organic matter content of <25 kgC/m^2^ based on de Vrese and Brovkin (*49*).

The CH_4_ is initially released where heterotrophic respiration occurs, especially in regions with high SOM contents and large inundated fractions (Figs. 2D and 3, A and D, and fig. S5). As the climate warms, ground ice melts, and surface water storage increases, the inundated fraction increases primarily in grid cells already partially saturated, which considerably overlap with the region dominated by organic soils (Figs. 2D and 3, B and C). As a result, the increase in CH_4_ emissions is concentrated in regions with significant increases in inundated areas and high SOM contents, i.e., in regions where CH_4_ emissions are originally high (Fig. 3, A to F).

Increased inundated fractions, mainly in organic soils, do not return to the previous state after the onset of net-zero and negative emissions, exhibiting irreversibility (Fig. 3G). Most regions with high SOM content overlap with peatland areas (fig. S5), which have low-lying topography that hinders natural runoff drainage. This geomorphological condition likely leads to an irreversible increase in inundation fractions that persists until the end of the simulation (Fig. 3). However, this needs to be verified through further studies. As a result, CH_4_ emissions remain relatively high in organic soils with permanently larger inundated fractions despite the decreased heterotrophic respiration (Fig. 3G and figs. S3 and S4). In Exp_neg, respiration declines by ~80% from the peak of CO_2_ to the year 2197, but CH_4_ emissions decrease relatively less by ~63%. These results suggest that under net-zero and negative emissions, the ratio of CH_4_ to CO_2_ may increase. As a result, the greenhouse gas forcing driven by permafrost degradation could increase due to the high global warming potential of CH_4_, placing additional burden on mitigation efforts (*44*, *48*).

### Hysteresis of near-surface permafrost and its carbon cycle

**Fig. 4.**
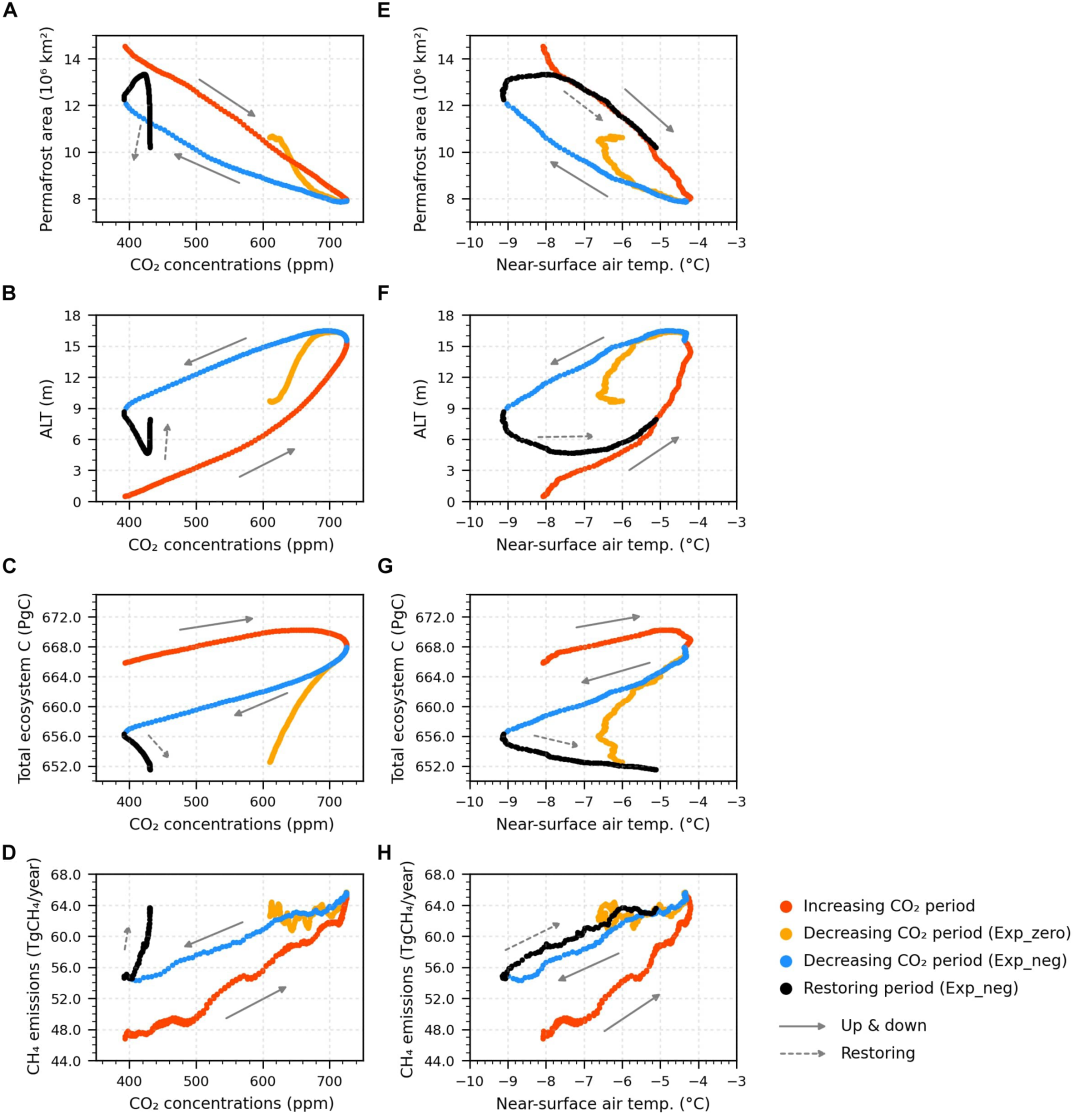
Hysteresis of permafrost response to changing CO_2_ forcing and SAT. (**A** and **E**) Permafrost area, (**B** and **F**) annual mean ALT, (**C** and **G**) annual mean total ecosystem carbon, and (**D** and **H**) annual CH_4_ emissions (A to D) as a function of CO_2_ concentrations and (E to H) SAT. Total ecosystem carbon is the sum of vegetation, litter, and soil carbon. All variables except for the permafrost area are averaged over the initial permafrost domain. All values are smoothed by the 11-year moving average. The dot colors indicate each period defined based on the evolution of atmospheric CO_2_ concentrations (pink: increasing CO_2_ period, orange and blue: decreasing CO_2_ period in Exp_zero and Exp_neg, black: restoring period in Exp_neg).

So far, we showed that the responses of the permafrost and its carbon cycle are not linear and strongly dependent on the pathway of the anthropogenic C emissions and climate conditions. This implies that the permafrost will show strong hysteresis behavior to CO_2_ forcing. First of all, the area of near-surface permafrost shows a hysteresis with respect to both CO_2_ concentration and SAT (Fig. 4, A and E). As the CO_2_ concentration decreases, the permafrost area recovers, but it is smaller at the same CO_2_ concentration and temperature, compared to the period of increasing CO_2_. In detail, in Exp_neg, the average of permafrost extent during the entire period of decreasing CO_2_ is 1.8 × 10^6^ km^2^ smaller than during the period of increasing CO_2_, despite the colder environment (Figs. 1B and 4A). Even at the same temperature, the permafrost area is much smaller during the decreasing CO_2_ period (Fig. 4E). The ALT also exhibits a hysteresis similar to that of permafrost extent but to a greater extent (Fig. 4, B and F). During the decreasing CO_2_ period, in Exp_neg, the ALT is on average ~1.5 times deeper (~3.8 m) than during the increasing CO_2_ period. These results suggest that permafrost continues to thaw to a greater depth during the decreasing CO_2_ period, which may continue to amplify the decomposability of deep soil C.

Eliseev *et al.* (*30*) showed that the slow recovery of permafrost in the cooling phase is related to the thermal inertia of soil water. The increased number of phase transition fronts due to the existence of talik (perennially thawed subsurface soils formed during the warming period) in the cooling phase further enhances the heat inertia of the soil column. The greater the organic carbon density, the greater the porosity and soil water, and thus the higher the thermal capacity in the soil column (*49*). In regions with organic-rich soils, the high organic content of soil enhances the thermal inertia and thus exhibits twice slower recovery compared to that in mineral soils (fig. S3). We note that permafrost extent and ALT recover slowly, but these are eventually reversible. Given enough time to adjust, they return to their previous state, as shown by the restoring period in Exp_neg.

Total terrestrial ecosystem C in the permafrost region, however, exhibits an open loop with respect to both CO_2_ and SAT (Fig. 4, C and G), suggesting the irreversible changes at least until the year 2300 under these mitigation pathways (*50*). The total ecosystem C increases during the positive emission phase. However, just before the CO_2_ concentration starts to decline, the total ecosystem carbon begins to decrease continuously until the end of the simulations. The total cumulative C emissions from permafrost until the end of the simulation are similar between the two scenarios. The changes in CH_4_ emissions also show an open loop, with higher emission rates after the cessation of positive emissions, suggesting a possibility of irreversible change (Fig. 4, D and H). This hysteresis behavior of the permafrost carbon cycle is observed in regions with organic-rich soils not in mineral soils (Fig. 4 and fig. S3). These results suggest that the slow recovery of permafrost, due to the thermal inertia of organic soils, contributes to hysteresis in the permafrost carbon cycle. This could create a long-lasting positive feedback to climate system, hindering climate mitigation efforts.

## DISCUSSION

In this study, we conducted idealized emission-driven simulations to investigate the response of permafrost under net-zero and negative emission scenarios. After the cessation of positive emissions, permafrost extent and ALT begin to recover but the recovery is slow compared to the rate of temperature decrease due to the thermal inertia of the soil, especially in regions with high SOM content. The total ecosystem C anomaly in the permafrost region is positive during the positive emissions phase due to an increase in vegetation C uptake that outweighs soil respiration. However, after the onset of the net-zero and negative emissions, vegetation C remains stable or decreases, while respiratory C loss from the litter-soil C pool gradually increases, leading to net ecosystem C loss in the permafrost region. This continues until the end of the simulations, ultimately leading to irreversible loss of ecosystem C in permafrost regions. Continued ecosystem C loss after the onset of net-zero and negative emissions could be a potential risk that partially reduces the effectiveness of CDR, thereby inhibiting climate change mitigation.

The total amount of ecosystem C loss in the negative emissions scenario is similar to that in the net-zero emissions scenario. This is because vegetation C is reduced due to decreased CO_2_ concentrations, and litter-soil C losses continue due to the slow recovery of permafrost under the negative emissions. In addition, even after the negative emissions cease, the AMOC overshoot causes surface warming, leading to further losses of litter-soil C. As a result, achieving negative emissions would not provide the additional benefit of preventing the loss of ecosystem C stored in permafrost.

Moreover, there is an irreversible change in CH_4_ emissions, with an increase during the net-zero and negative emission phases compared to the positive emission phase. This is because even if the permafrost and ALT partially recover, the increased inundated fractions in organic soils due to permafrost degradation do not return to their original state.

In summary, net ecosystem C loss in the permafrost begins after the cessation of positive emissions and continues until the end of the experiment, with the same amount of release of ~14 PgC in both experiments. In addition, CH_4_ emissions increase after the cessation of positive emissions, resulting in higher greenhouse gas forcing in the permafrost region. These results suggest that, contrary to our expectations, even if net-zero and negative emissions are achieved, permafrost may pose a considerable risk in efforts to mitigate climate change. Therefore, these changes in permafrost should be considered in long-term climate mitigation policies.

At the current state, our projections of permafrost ecosystem C emissions under net-zero and negative emissions contain uncertainties regarding changes in the permafrost and Arctic ecosystem and the AMOC-induced climate change. First, the amount of soil C emissions is uncertain due to poor understanding of decomposition dynamics in deep soils and soil C content, as well as incomplete representation of permafrost processes in the model (*28*, *51*, *52*). Permafrost soil C pool simulated in CLM5 differs from field-based estimates or site-level observations and is unrealistically high in some grid cells due to the artificial initialization procedure (*29*, *31*, *53*). In addition, the physical mixing of soil C by cryoturbation is simply represented by a diffusion equation proportional to the vertical gradient of soil C (see Materials and Methods). Therefore, during the positive emission period, high SOM gradient caused by increased litter fall could have potentially led to unnaturally fast rates of carbon burial into deep soil layers. An abrupt thawing in ice-rich permafrost, formation of thermokarst bog, impact of wildfires on permafrost thaw, and their impacts on soil C are not represented here, which may lead to considerable underestimation of future permafrost soil C loss (*46*, *54*–*58*). In addition, CH_4_ emissions and their sensitivity to climate change are still uncertain due to the complexity of processes related to CH_4_ biogeochemistry and interaction with soil moisture (*42*, *44*).

Second, many processes related to Arctic ecosystems are still poorly represented: simple vegetation community and static vegetation composition (*28*, *43*, *59*). This may underestimate future C uptake by plants and also neglect the inertia effect of vegetation under net-zero or negative emissions.

Last, although most ESMs simulate similar behavior of the AMOC in overshoot scenarios or CO_2_ ramp-up and ramp-down experiments, CESM2 shows a relatively strong decline and recovery of the AMOC (*10*, *31*, *34*). This could lead to relatively strong temperature fluctuations in the Northern Hemisphere, with potentially distinctive impacts on permafrost and ecosystem processes. Therefore, there is a need for more accurate reestimation of changes in permafrost ecosystem C in the future through the improvement of model processes. In addition, it is important to assess the range of uncertainty in a multimodel framework, with efforts to include permafrost processes in other ESMs, and to consider this in long-term climate mitigation strategies.

## MATERIALS AND METHODS

### Model configuration

In this study, we used the CESM2, which comprises the atmosphere (The Community Atmosphere Model version 6), ocean (The Parallel Ocean Program version 2), sea ice (The Community Ice CodE version 5), and land models (CLM5) (*35*). CLM5 is capable of simulating the key thermal, hydrological, and biogeochemical processes associated with permafrost and their response to climate warming (*60*). CLM5 has improved its components, such as soil layer resolution, vertically resolved soil biogeochemistry, soil hydrology in cold regions, and snow density and insulation for a more realistic simulation of active layer dynamics, permafrost distribution, and hydrology (*60*).

In permafrost-affected soils, the vertical mixing of soil C is accomplished by the cryoturbation scheme proposed by Koven *et al.* (*61*), which uses a simple diffusion equation

$$\frac{\partial C_{i}}{\partial t}=D\frac{\partial^{2}C_{i}}{\partial z^{2}}$$

where *C_i_* is the *C* concentration of *C* pool *i*, and D is the cryoturbation diffusive parameter (10^−3^m^2^y^−1^), which yields a mixing timescale of approximately 1000 years over the top meter of soil. The diffusivity parameter, *D*, is constant within the active layer but tapers linearly to zero at a depth of three times the ALT. This cryoturbation scheme allows soil C, generated near the surface, to move downward into deeper soil layers, leading to better agreement with estimates of high-latitude soil C stocks, where substantial amounts of carbon are found in permafrost regions, stored to depths of 3 m (*61*, *62*).

The CLM5 also includes a methane biogeochemical model, which represents a complex and interacting series of processes to simulate net surface CH_4_ emissions: the production of CH_4_ in anaerobic conditions, oxidation within the soil column, aerenchyma transport, ebullition, aqueous and gaseous diffusion, and fractional inundation (*42*–*44*). Because CLM5 does not specifically simulate the wetland fraction or related soil biogeochemical processes, heterotrophic respiration is used to estimate the wetland decomposition rate. In each grid cell, the equations for CH_4_ and O_2_ are solved separately for the inundated and non-inundated fractions. Inundation fraction is calculated using water table depth and microtopographic distribution, normally distributed around the grid cell mean elevation. We note that changes in CH_4_ emissions do not account for changes in wetland or lake distribution that could occur due to thermokarst formation or other thaw-related landscape dynamics (*42*, *46*, *54*). We also note that the CLM5 does not include dynamic vegetation biogeography, and vegetation distributions are prescribed and held at the present-day condition (year 2000) (*43*).

### Experimental design

To avoid discontinuity, we designed two idealized emission pathways, assuming a gradual reduction of emissions through the continuous development of CDR technology. They are subdivided based on whether net-zero (Exp_zero) or negative emissions (Exp_neg) are implemented immediately after the positive emissions (Fig. 1A and table S1). In both experiments, anthropogenic CO_2_ emissions increase linearly from 2000 to 2050, based on the SSP5-8.5 scenario (1.09 GtCO_2_ increase per year), and then decrease at the same rate until 2123 (~1500 PgC of total cumulative emissions). Thereafter, in Exp_zero, net anthropogenic CO_2_ emissions are held at zero until the end of the simulation (year 2300), whereas in Exp_neg, CO_2_ emissions gradually decline at the same rate until the global mean surface atmospheric CO_2_ concentration recovers to its initial value (~383 ppm; until year 2197): ~800 PgC of cumulative artificial CO_2_ removal is required. Immediately after the initial recovery of CO_2_ concentrations, net-zero emissions are maintained in Exp_neg until the end of the simulation.

On the basis of these scenarios, we performed simulations in emission-driven mode. Atmospheric CO_2_ concentrations are determined not only by prescribed anthropogenic CO_2_ emissions but also by terrestrial and oceanic CO_2_ fluxes, thereby reflecting changes in CO_2_ concentration through the carbon cycle-climate feedback. In Exp_zero, the atmospheric CO_2_ level peaks decades before the onset of net-zero emissions (year 2107) when anthropogenic emissions become equal to natural C uptake and then begin to gradually decline due to continuous natural C uptake (*10*, *63*). For a detailed discussion of the evolution of CO_2_ concentrations during the negative emission phases, we refer to Park *et al.* (*41*). Here, we focus only on the permafrost response to net-zero and negative emissions. All non-CO_2_ conditions, such as land use and non-CO_2_ greenhouse gas forcings, are kept at the present-day (2000) level. We have run a total of seven ensemble members with slightly different initial conditions for each experiment.

## Supplementary Materials

### This PDF file includes:

Table S1 Figs. S1 to S5 References

## References

[1] M. R. Allen, D. J. Frame, C. Huntingford, C. D. Jones, J. A. Lowe, M. Meinshausen, N. Meinshausen, Warming caused by cumulative carbon emissions towards the trillionth tonne. Nature 458, 1163–1166 (2009). 19407800 10.1038/nature08019

[2] T. A. Carleton, S. M. Hsiang, Social and economic impacts of climate. Science 353, aad9837 (2016). 27609899 10.1126/science.aad9837

[3] IPCC, Long-term climate change: Projections, commitments and irreversibility, in *Climate Change 2013: The Physical Science Basis. Contribution of Working Group I to the Fifth Assessment Report of the Intergovernmental Panel on Climate Change*, T. F. Stocker, D. Qin, G.-K. Plattner, M. Tignor, S. K. Allen, J. Boschung, A. Nauels, Y. Xia, V. Bex, P. M. Midgley, Eds. (Cambridge Univ. Press, 2013), pp. 1029–1136.

[4] H. Matthews, N. Gillett, P. Stott, K. Zickfeld, The proportionality of global warming to cumulative carbon emissions. Nature 459, 829–832 (2009). 19516338 10.1038/nature08047

[5] United Nations Framework on Climate Change (UNFCCC), Adoption of the Paris Agreement, 21st Conference of the Parties (2015); http://unfccc.int/resource/docs/2015/cop21/eng/l09r01.pdf.

[6] T. Gasser, C. Guivarch, K. Tachiiri, C. D. Jones, P. Ciais, Negative emissions physically needed to keep global warming below 2°C. Nat. Commun. 6, 7958 (2015). 26237242 10.1038/ncomms8958

[7] E. Kriegler, G. Luderer, N. Bauer, L. Baumstark, S. Fujimori, A. Popp, J. Rogelj, J. Strefler, D. P. van Vuuren, Pathways limiting warming to 1.5°C: A tale of turning around in no time? Philos. Trans. A Math. Phys. Eng. Sci. 376, 20160457 (2018). 29610367 10.1098/rsta.2016.0457 PMC5897828

[8] J. Rogelj, M. Schaeffer, P. Friedlingstein, N. P. Gillett, D. P. Van Vuuren, K. Riahi, M. Allen, R. Knutti, Differences between carbon budget estimates unravelled. Nat. Clim. Change 6, 245–252 (2016).

[9] IPCC, 2018: Summary for Policymakers in *Global Warming of 1.5°C. An IPCC Special Report on the impacts of global warming of 1.5°C above pre-industrial levels and related global greenhouse gas emission pathways, in the context of strengthening the global response to the threat of climate change, sustainable development, and efforts to eradicate poverty*, Masson-Delmotte, V., P. Zhai, H.-O. Pörtner, D. Roberts, J. Skea, P. R. Shukla, A. Pirani, W. Moufouma-Okia, C. Péan, R. Pidcock, S. Connors, J. B. R. Matthews, Y. Chen, X. Zhou, M. I. Gomis, E. Lonnoy, T. Maycock, M. Tignor, and T. Waterfield, Eds. (Cambridge Univ. Press, 2022), pp. 3–24; 10.1017/9781009157940.001.

[10] J. Schwinger, A. Asaadi, N. J. Steinert, H. Lee, Emit now, mitigate later? Earth system reversibility under overshoots of different magnitudes and durations. Earth Syst. Dynam. 13, 1641–1665 (2022).

[11] O. Boucher, P. R. Halloran, E. J. Burke, M. Doutriaux-Boucher, C. D. Jones, J. Lowe, M. A. Ringer, E. Robertson, P. Wu, Reversibility in an Earth System model in response to CO_2_ concentration changes. Environ. Res. Lett. 7, 024013 (2012).

[12] L. Cao, G. Bala, K. Caldeira, Why is there a short-term increase in global precipitation in response to diminished CO_2_ forcing? Geophys. Res. Lett. 38, L06703 (2011).

[13] T. L. Frölicher, F. Joos, Reversible and irreversible impacts of greenhouse gas emissions in multi-century projections with the NCAR global coupled carbon cycle-climate model. Clim. Dyn. 35, 1439–1459 (2010).

[14] A. Jeltsch-thömmes, T. F. Stocker, F. Joos, Hysteresis of the Earth system under positive and negative CO_2_ emissions. Environ. Res. Lett. 15, 124026 (2020).

[15] P. Wu, J. Ridley, A. Pardaens, R. Levine, J. Lowe, The reversibility of CO_2_ induced climate change. Clim. Dyn. 45, 745–754 (2015).

[16] K. B. Tokarska, K. Zickfeld, The effectiveness of net negative carbon dioxide emissions in reversing anthropogenic climate change. Environ. Res. Lett. 10, 094013 (2015).

[17] A. H. MacDougall, Reversing climate warming by artificial atmospheric carbon-dioxide removal: Can a Holocene-like climate be restored? Geophys. Res. Lett. 40, 5480–5485 (2013).

[18] C. Voigt, M. E. Marushchak, B. W. Abbott, C. Biasi, B. Elberling, S. D. Siciliano, O. Sonnentag, K. J. Stewart, Y. Yang, P. J. Martikainen, Nitrous oxide emissions from permafrost-affected soils. Nat. Rev. Earth Environ. 1, 420–434 (2020).

[19] E. A. G. Schuur, B. W. Abbott, R. Commane, J. Ernakovich, E. Euskirchen, G. Hugelius, G. Grosse, M. Jones, C. Koven, V. Leshyk, D. Lawrence, M. M. Loranty, M. Mauritz, D. Olefeldt, S. Natali, H. Rodenhizer, V. Salmon, C. Schädel, J. Strauss, C. Treat, M. Turetsky, Permafrost and climate change: Carbon cycle feedbacks from the warming arctic. Annu. Rev. Env. Resour. 47, 343–371 (2022).

[20] A. H. MacDougall, K. Zickfeld, R. Knutti, H. D. Matthews, Sensitivity of carbon budgets to permafrost carbon feedbacks and non-CO_2_ forcings. Environ. Res. Lett. 10, 125003 (2015).

[21] E. A. G. Schuur, J. Bockheim, J. G. Canadell, E. Euskirchen, C. B. Field, S. V. Goryachkin, S. Hagemann, P. Kuhry, P. M. Lafleur, H. Lee, G. Mazhitova, F. E. Nelson, A. Rinke, V. E. Romanovsky, N. Shiklomanov, C. Tarnocai, S. Venevsky, J. G. Vogel, S. A. Zimov, Vulnerability of permafrost carbon to climate change: Implications for the global carbon cycle. Bioscience 58, 701–714 (2008).

[22] E. A. G. Schuur, A. D. McGuire, C. Schädel, G. Grosse, J. W. Harden, D. J. Hayes, G. Hugelius, C. D. Koven, P. Kuhry, D. M. Lawrence, S. M. Natali, D. Olefeldt, V. E. Romanovsky, K. Schaefer, M. R. Turetsky, C. C. Treat, J. E. Vonk, Climate change and the permafrost carbon feedback. Nature 520, 171–179 (2015). 25855454 10.1038/nature14338

[23] E. J. Burke, A. Ekici, Y. Huang, S. E. Chadburn, C. Huntingford, P. Ciais, P. Friedlingstein, S. Peng, G. Krinner, Quantifying uncertainties of permafrost carbon–climate feedbacks. Biogeosciences 14, 3051–3066 (2017).

[24] J. A. Lowe, D. Bernie, The impact of Earth system feedbacks on carbon budgets and climate response. Philos. Trans. R. Soc. A 376, 20170263 (2018). 10.1098/rsta.2017.0263 PMC5897833 29610375

[25] K. Schaefer, H. Lantuit, V. E. Romanovsky, E. A. G. Schuur, R. Witt, The impact of the permafrost carbon feedback on global climate. Environ. Res. Lett. 9, 085003 (2014).

[26] K. R. Miner, M. R. Turetsky, E. Malina, A. Bartsch, J. Tamminen, A. D. McGuire, A. Fix, C. Sweeney, C. D. Elder, C. E. Miller, Permafrost carbon emissions in a changing Arctic. Nat. Rev. Earth Environ. 3, 55–67 (2022).

[27] Z. Zhu, S. Piao, R. B. Myneni, M. Huang, Z. Zeng, J. G. Canadell, P. Ciais, S. Sitch, P. Friedlingstein, A. Arneth, C. Cao, L. Cheng, E. Kato, C. Koven, Y. Li, X. Lian, Y. Liu, R. Liu, J. Mao, Y. Pan, S. Peng, J. Peñuelas, B. Poulter, T. A. M. Pugh, B. D. Stocker, N. Viovy, X. Wang, Y. Wang, Z. Xiao, H. Yang, S. Zaehle, N. Zeng, Greening of the Earth and its drivers. Nat. Clim. Change 6, 791–795 (2016).

[28] C. D. Koven, D. M. Lawrence, W. J. Riley, Permafrost carbon−climate feedback is sensitive to deep soil carbon decomposability but not deep soil nitrogen dynamics. Proc. Natl. Acad. Sci. U.S.A. 112, 3752–3757 (2015). 25775603 10.1073/pnas.1415123112 PMC4378430

[29] S.-W. Park, J.-S. Kug, A decline in atmospheric CO_2_ levels under negative emissions may enhance carbon retention in the terrestrial biosphere. Commun. Earth Environ. 3, 289 (2022).

[30] A. V. Eliseev, P. F. Demchenko, M. M. Arzhanov, I. I. Mokhov, Transient hysteresis of near-surface permafrost response to external forcing. Clim. Dyn. 42, 1203–1215 (2014).

[31] C. D. Koven, V. K. Arora, P. Cadule, R. A. Fisher, C. D. Jones, D. M. Lawrence, J. Lewis, K. Lindsay, S. Mathesius, M. Meinshausen, M. Mills, Z. Nicholls, B. M. Sanderson, R. Séférian, N. C. Swart, W. R. Wieder, K. Zickfeld, Multi-century dynamics of the climate and carbon cycle under both high and net negative emissions scenarios. Earth Syst. Dynam. 13, 885–909 (2022).

[32] C. D. Jones, V. Arora, P. Friedlingstein, L. Bopp, V. Brovkin, J. Dunne, H. Graven, F. Hoffman, T. Ilyina, J. G. John, M. Jung, M. Kawamiya, C. Koven, J. Pongratz, T. Raddatz, J. T. Randerson, S. Zaehle, C4MIP–The Coupled Climate–Carbon Cycle Model Intercomparison Project: Experimental protocol for CMIP6. Geosci. Model Dev. 9, 2853–2880 (2016).

[33] C. D. Koven, B. M. Sanderson, A. L. S. Swann, Much of zero emissions commitment occurs before reaching net zero emissions. Environ. Res. Lett. 18, 014017 (2023).

[34] J. Schwinger, A. Asaadi, N. Goris, H. Lee, Possibility for strong northern hemisphere high-latitude cooling under negative emissions. Nat. Commun. 13, 1095 (2022). 35232955 10.1038/s41467-022-28573-5 PMC8888562

[35] G. Danabasoglu, J.-F. Lamarque, J. Bacmeister, D. A. Bailey, A. K. DuVivier, J. Edwards, L. K. Emmons, J. Fasullo, R. Garcia, A. Gettelman, C. Hannay, M. M. Holland, W. G. Large, P. H. Lauritzen, D. M. Lawrence, J. T. M. Lenaerts, K. Lindsay, W. H. Lipscomb, M. J. Mills, R. Neale, K. W. Oleson, B. Otto-Bliesner, A. S. Phillips, W. Sacks, S. Tilmes, L. van Kampenhout, M. Vertenstein, A. Bertini, J. Dennis, C. Deser, C. Fischer, B. Fox-Kemper, J. E. Kay, D. Kinnison, P. J. Kushner, V. E. Larson, M. C. Long, S. Mickelson, J. K. Moore, E. Nienhouse, L. Polvani, P. J. Rasch, W. G. Strand, The Community Earth System Model Version 2 (CESM2). J. Adv. Model. Earth Syst. 12, e2019MS001916 (2020).

[36] N. Steinert, M. Debolskiy, E. Burke, F. García-Pereira, H. Lee, Evaluating permafrost definitions for global permafrost area estimates in CMIP6 climate models. Environ. Res. Lett. 19, 014033 (2023).

[37] S.-I. An, J. Shin, S.-W. Yeh, S.-W. Son, J.-S. Kug, S.-K. Min, H.-J. Kim, Global cooling hiatus driven by an AMOC overshoot in a carbon dioxide removal scenario. Earths Future *9*, e2021EF002165 (2021).

[38] J.-S. Kug, J.-H. Oh, S.-I. An, S.-W. Yeh, S.-K. Min, S.-W. Son, J. Kam, Y.-G. Ham, J. Shin, Hysteresis of the intertropical convergence zone to CO_2_ forcing. Nat. Clim. Change 12, 47–53 (2022).

[39] H. Lee, A. Ekici, J. Tjiputra, H. Muri, S. E. Chadburn, D. M. Lawrence, J. Schwinger, The response of permafrost and high-latitude ecosystems under large-scale stratospheric aerosol injection and its termination. Earths Future 7, 605–614 (2019).

[40] G. B. Bonan, *Climate Change and Terrestrial Ecosystem Modeling* (Cambridge Univ. Press, 2019).

[41] S.-W. Park, K. M. Noh, S.-I. An, J. Kam, E.-Y. Kwon, S.-K. Min, R. Park, S.-W. Son, S.-W. Yeh, J.-S. Kug, How will global carbon cycle respond to negative emissions? ESS Open Archive (2023).

[42] W. J. Riley, Z. M. Subin, D. M. Lawrence, S. C. Swenson, M. S. Torn, L. Meng, N. M. Mahowald, P. Hess, Barriers to predicting changes in global terrestrial methane fluxes: Analyses using CLM4Me, a methane biogeochemistry model integrated in CESM. Biogeosciences. 8, 1925–1953 (2011).

[43] D. Lawrence, R. Fisher, C. Koven, K. Oleson, S. Swenson, M. Vertenstein, B. Andre, G. Bonan, B. Ghimire, L. van Kampenhout, D. Kennedy, E. Kluzek, R. Knox, P. Lawrence, F. Li, H. Li, D. Lombardozzi, Y. Lu, J. Perket, W. Riley, W. Sacks, M. Shi, W. Wieder, C. Xu, A. Ali, A. Badger, G. Bisht, P. Broxton, M. Brunke, J. Buzan, M. Clark, T. Craig, K. Dahlin, B. Drewniak, L. Emmons, J. Fisher, M. Flanner, P. Gentine, J. Lenaerts, S. Levis, Technical Description of version 5.0 of the Community Land Model (CLM) [National Center for Atmospheric Research (NCAR), 2018].

[44] D. M. Lawrence, C. D. Koven, S. C. Swenson, W. J. Riley, A. G. Slater, Permafrost thaw and resulting soil moisture changes regulate projected high-latitude CO_2_ and CH_4_ emissions. Environ. Res. Lett. 10, 94011 (2015).

[45] D. Olefeldt, M. R. Turetsky, P. M. Crill, A. D. McGuire, Environmental and physical controls on northern terrestrial methane emissions across permafrost zones. Glob. Change Biol. 19, 589–603 (2013). 10.1111/gcb.12071 23504795

[46] H. Lee, S. C. Swenson, A. G. Slater, D. M. Lawrence, Effects of excess ground ice on projections of permafrost in a warming climate. Environ. Res. Lett. 9, 124006 (2014).

[47] C. C. Treat, S. M. Natali, J. Ernakovich, C. M. Iversen, M. Lupascu, A. D. McGuire, R. J. Norby, T. Roy Chowdhury, A. Richter, H. Šantrůčková, C. Schädel, E. A. G. Schuur, V. L. Sloan, M. R. Turetsky, M. P. Waldrop, A pan-Arctic synthesis of CH_4_ and CO_2_ production from anoxic soil incubations. Glob. Change Biol. 21, 2787–2803 (2015). 10.1111/gcb.12875 25620695

[48] J. Huang, B. Mendoza, J. S. Daniel, C. J. Nielsen, L. Rotstayn, O. Wild, Anthropogenic and natural radiative forcing, *Clim. Chang. 2013 Phys. Sci. Basis Work. Gr. I Contrib. to Fifth Assess. Rep. Intergov. Panel Clim. Chang.* (Cambridge Univ. Press, 2013), pp. 659–740, 9781107057999.

[49] P. de Vrese, V. Brovkin, Timescales of the permafrost carbon cycle and legacy effects of temperature overshoot scenarios. Nat. Commun. 12, 2688 (2021). 33976172 10.1038/s41467-021-23010-5 PMC8113593

[50] S.-K. Kim, J. Shin, S.-I. An, H.-J. Kim, N. Im, S.-P. Xie, J.-S. Kug, S.-W. Yeh, Widespread irreversible changes in surface temperature and precipitation in response to CO_2_ forcing. Nat. Clim. Change 12, 834–840 (2022).

[51] G. Hugelius, J. Strauss, S. Zubrzycki, J. W. Harden, E. A. G. Schuur, C.-L. Ping, L. Schirrmeister, G. Grosse, G. J. Michaelson, C. D. Koven, J. A. O’Donnell, B. Elberling, U. Mishra, P. Camill, Z. Yu, J. Palmtag, P. Kuhry, Estimated stocks of circumpolar permafrost carbon with quantified uncertainty ranges and identified data gaps. Biogeosciences 11, 6573–6593 (2014).

[52] G. Hugelius, J. Loisel, S. Chadburn, R. B. Jackson, M. Jones, G. MacDonald, M. Marushchak, D. Olefeldt, M. Packalen, M. B. Siewert, C. Treat, M. Turetsky, C. Voigt, Z. Yu, Large stocks of peatland carbon and nitrogen are vulnerable to permafrost thaw. Proc. Natl. Acad. Sci. U.S.A. 117, 20438–20446 (2020). 32778585 10.1073/pnas.1916387117 PMC7456150

[53] E. J. Burke, Y. Zhang, G. Krinner, Evaluating permafrost physics in the Coupled Model Intercomparison Project 6 (CMIP6) models and their sensitivity to climate change. Cryosphere 14, 3155–3174 (2020).

[54] A. Ekici, H. Lee, D. M. Lawrence, S. C. Swenson, C. Prigent, Ground subsidence effects on simulating dynamic high-latitude surface inundation under permafrost thaw using CLM5. Geosci. Model Dev. 12, 5291–5300 (2019).

[55] M. R. Turetsky, B. W. Abbott, M. C. Jones, K. W. Anthony, D. Olefeldt, E. A. G. Schuur, G. Grosse, P. Kuhry, G. Hugelius, C. Koven, D. M. Lawrence, C. Gibson, A. B. K. Sannel, A. D. McGuire, Carbon release through abrupt permafrost thaw. Nat. Geosci. 13, 138–143 (2020).

[56] J. Nitzbon, T. Schneider von Deimling, M. Aliyeva, S. Chadburn, G. Grosse, S. Laboor, H. Lee, G. Lohmann, N. Steinert, S. Stuenzi, M. Werner, S. Westermann, M. Langer, No respite from permafrost-thaw impacts in absence of a global tipping point. Nat. Clim. Change 14, 573–585 (2023).

[57] C. M. Gibson, L. E. Chasmer, D. K. Thompson, W. L. Quinton, M. D. Flannigan, D. Olefeldt, Wildfire as a major driver of recent permafrost thaw in boreal peatlands. Nat. Commun. 9, 3041 (2018). 30072751 10.1038/s41467-018-05457-1 PMC6072743

[58] K. Van Huissteden, *Thawing Permafrost: Permafrost Carbon in a Warming Arctic* (Springer, 2020).

[59] J. B. Fisher, M. Sikka, W. C. Oechel, D. N. Huntzinger, J. R. Melton, C. D. Koven, A. Ahlström, M. A. Arain, I. Baker, J. M. Chen, P. Ciais, C. Davidson, M. Dietze, B. El-Masri, D. Hayes, C. Huntingford, A. K. Jain, P. E. Levy, M. R. Lomas, B. Poulter, D. Price, A. K. Sahoo, K. Schaefer, H. Tian, E. Tomelleri, H. Verbeeck, N. Viovy, R. Wania, N. Zeng, C. E. Miller, Carbon cycle uncertainty in the Alaskan Arctic. Biogeosciences 11, 4271–4288 (2014).

[60] D. M. Lawrence, R. A. Fisher, C. D. Koven, K. W. Oleson, S. C. Swenson, G. Bonan, N. Collier, B. Ghimire, L. van Kampenhout, D. Kennedy, E. Kluzek, P. J. Lawrence, F. Li, H. Li, D. Lombardozzi, W. J. Riley, W. J. Sacks, M. Shi, M. Vertenstein, W. R. Wieder, C. Xu, A. A. Ali, A. M. Badger, G. Bisht, M. van den Broeke, M. A. Brunke, S. P. Burns, J. Buzan, M. Clark, A. Craig, K. Dahlin, B. Drewniak, J. B. Fisher, M. Flanner, A. M. Fox, P. Gentine, F. Hoffman, G. Keppel-Aleks, R. Knox, S. Kumar, J. Lenaerts, L. R. Leung, W. H. Lipscomb, Y. Lu, A. Pandey, J. D. Pelletier, J. Perket, J. T. Randerson, D. M. Ricciuto, B. M. Sanderson, A. Slater, Z. M. Subin, J. Tang, R. Q. Thomas, M. Val Martin, X. Zeng, The Community Land Model Version 5: Description of new features, benchmarking, and impact of forcing uncertainty. J. Adv. Model Earth Syst. 11, 4245–4287 (2019).

[61] C. Koven, P. Friedlingstein, P. Ciais, D. Khvorostyanov, G. Krinner, C. Tarnocai, On the formation of high-latitude soil carbon stocks: Effects of cryoturbation and insulation by organic matter in a land surface model. Geophys. Res. Lett. 36, L21501 (2009).

[62] J. G. Bockheim, I. B. Campbell, M. McLeod, Permafrost distribution and active-layer depths in the McMurdo Dry Valleys, Antarctica. Permafr. Periglac. Process. 18, 217–227 (2007).

[63] A. H. MacDougall, T. L. Frölicher, C. D. Jones, J. Rogelj, H. D. Matthews, K. Zickfeld, V. K. Arora, N. J. Barrett, V. Brovkin, F. A. Burger, M. Eby, A. V. Eliseev, T. Hajima, P. B. Holden, A. Jeltsch-Thömmes, C. Koven, N. Mengis, L. Menviel, M. Michou, I. I. Mokhov, A. Oka, J. Schwinger, R. Séférian, G. Shaffer, A. Sokolov, K. Tachiiri, J. Tjiputra, A. Wiltshire, T. Ziehn, Is there warming in the pipeline? A multi-model analysis of the Zero Emissions Commitment from CO_2_. Biogeosciences 17, 2987–3016 (2020).

[64] D. M. Olson, E. Dinerstein, E. D. Wikramanayake, N. D. Burgess, G. V. N. Powell, E. C. Underwood, J. A. D’amico, I. Itoua, H. E. Strand, J. C. Morrison, C. J. Loucks, T. F. Allnutt, T. H. Ricketts, Y. Kura, J. F. Lamoreux, W. W. Wettengel, P. Hedao, K. R. Kassem, Terrestrial ecoregions of the world: A new map of life on Earth: A new global map of terrestrial ecoregions provides an innovative tool for conserving biodiversity. Bioscience 51, 933–938 (2001).

[65] C. Tarnocai, I. M. Kettles, B. Lacelle, Peatlands of Canada, Geological Survey of Canada, Open File 6561, CD-ROM. (2011).

[66] B. Lehner, P. Döll, Development and validation of a global database of lakes, reservoirs and wetlands. J. Hydrol. 296, 1–22 (2004).

