# Supplementary material for: Continued permafrost ecosystem carbon loss under net-zero and negative emissions
Source: Sci Adv. 2025 Feb 12;11(7):eadn8819. doi: 10.1126/sciadv.adn8819 (PMC11818019; doi:10.1126/sciadv.adn8819)
Supplement: Supplementary file 1 — Table S1 Figs. S1 to S5 References [file sciadv.adn8819_sm.pdf]

Science Advances
AAAS

Supplementary Materials for

Continued permafrost ecosystem carbon loss under net-zero and negative emissions

So-Won Park *et al.*

Corresponding author: Jong-Seong Kug, jskug1@gmail.com

Sci. Adv. **11**, eadn8819 (2025)
DOI: 10.1126/sciadv.adn8819

This PDF file includes:

Table S1
Figs. S1 to S5
References

Table S1. Overview of simulations involving net-zero (Exp_zero) or negative emissions (Exp_neg) immediately after positive emissions

(Exp_zero/Exp_neg)	Period1 (2001-2123)	Period 2 (2124-2196)	Period 3 (2197-2300)
Cumulative positive emissions	1506 Pg C	- / -	- / -
Cumulative CDR	-	- / 793 Pg C	- / -
Average CO_2 concentration	593 ppm	671 ppm / 575 ppm	624 ppm / 422 ppm
Difference of CO_2 (end– start year)	317 ppm	−65 ppm / −327 ppm	−34 ppm / 48 ppm

Each period is defined based on the evolution of anthropogenic CO_2 emissions (Period 1: positive emissions, Period 2: net-zero emissions in Exp_zero and negative emissions in Exp_neg, Period 3: net-zero emissions).

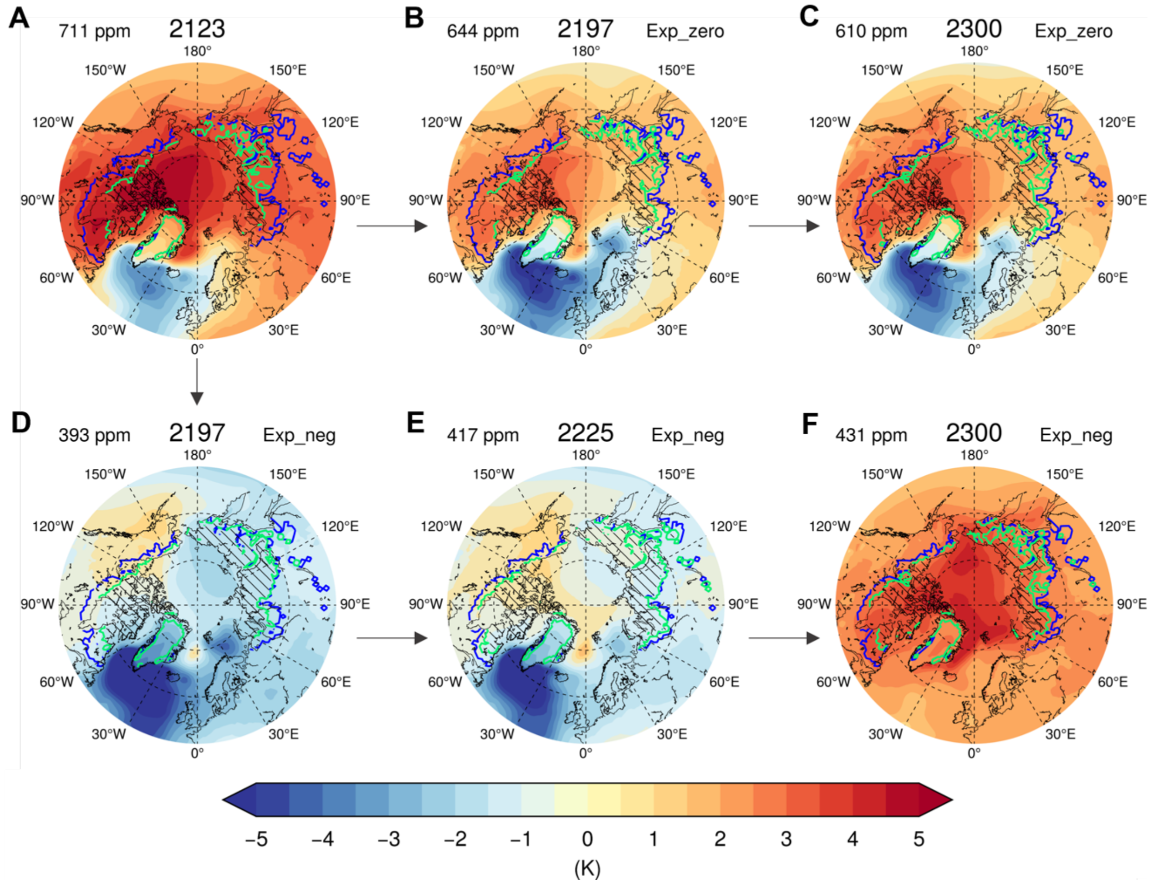

Fig. S1. **Changes in near-surface air temperature (SAT) and permafrost boundary. (A)** Annual mean changes in SAT at the year 2123 (end of positive emissions), **(B,D)** 2197 (end of negative emissions in Exp_neg), **(E)** 2225, and **(C,F)** 2300 (end of Exp_zero and Exp_neg) relative to year 2000 **(A-C)** in Exp_zero and **(A,D-F)** Exp_neg. All values are ensemble mean and smoothed by the 11-year moving average. The initial permafrost boundary is superimposed in blue. The simulated permafrost boundary for each period is superimposed in green. Hatched areas indicate the permafrost domain for each period.

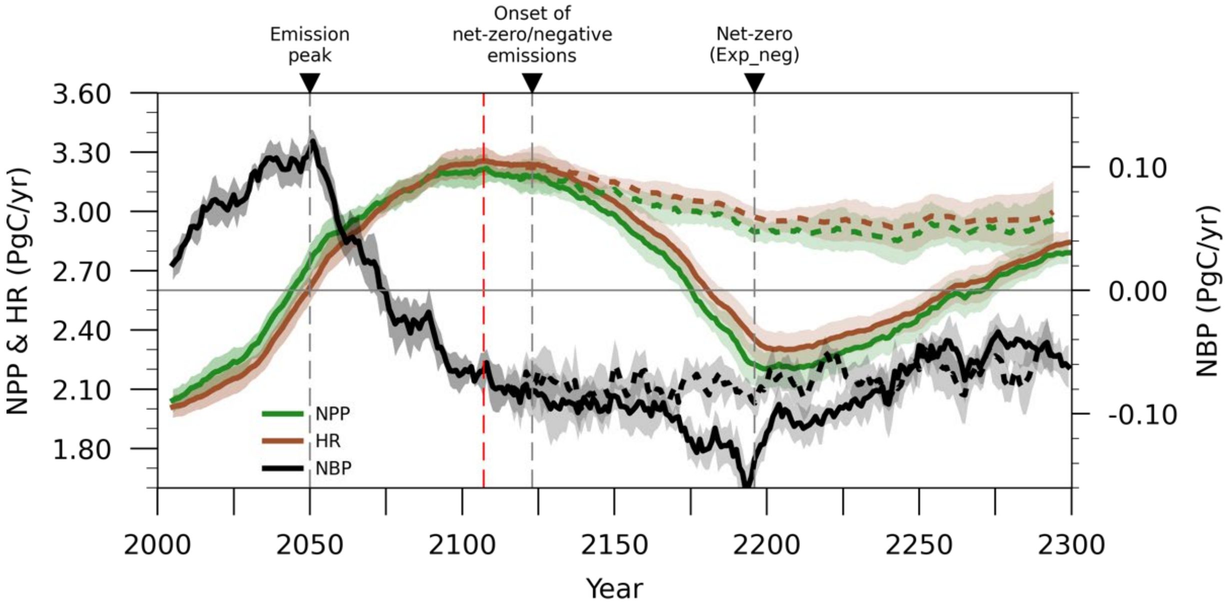

Fig. S2. Time-series of the 11-year running mean of annual net primary production (NPP), heterotrophic respiration (HR), and net biome productivity (NBP). The solid and dashed lines show the ensemble mean values for Exp_neg and Exp_zero, respectively. The shading indicates the 95% confidence interval based on the bootstrap method. All values are averaged over the initial permafrost domain and smoothed by the 11-year moving average. The peak of anthropogenic emissions and the onset of negative emissions are indicated by the gray dashed vertical line. The point of maximum CO_2 levels is indicated by the red dashed vertical line.

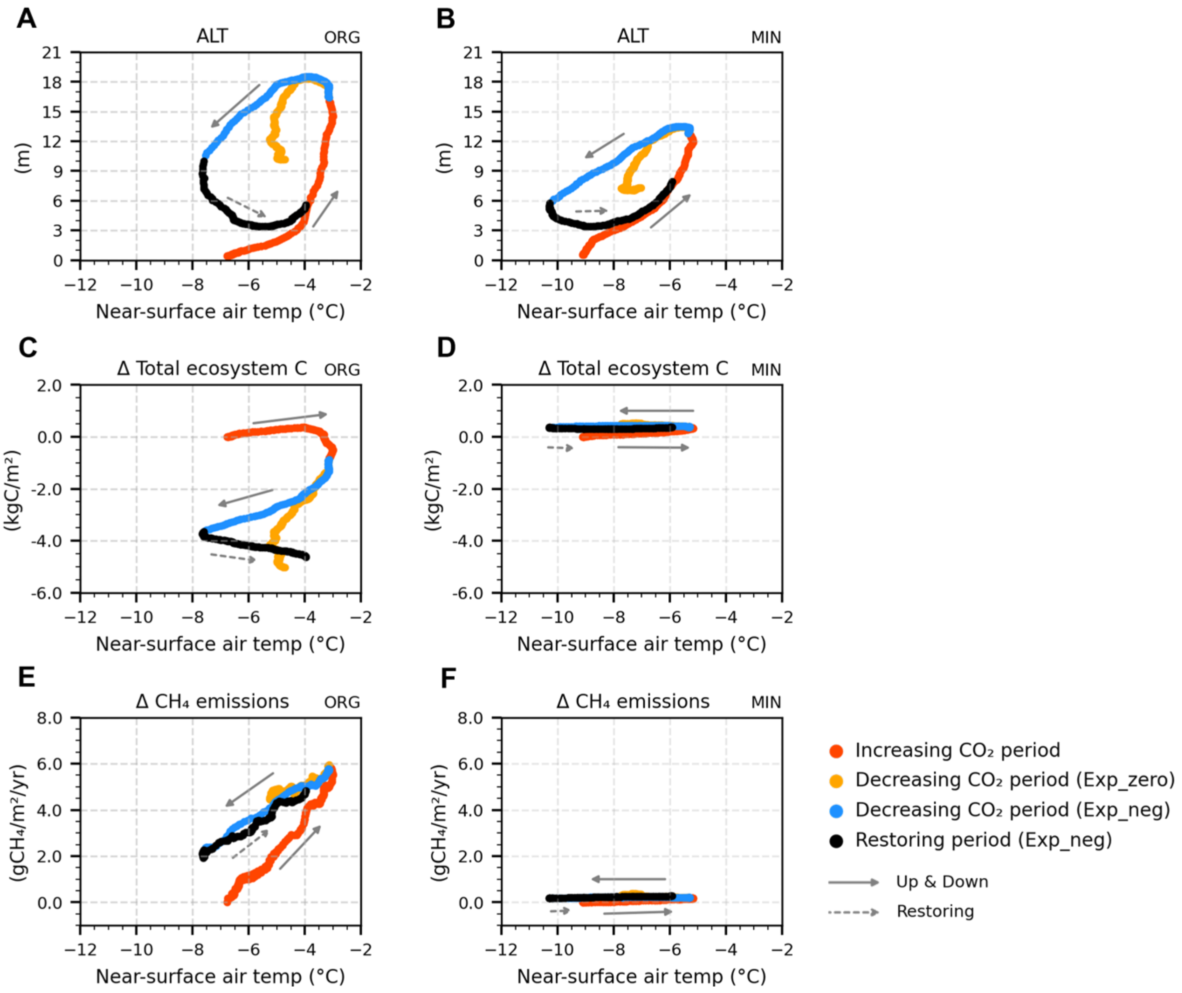

Fig. S3. Hysteresis of permafrost area and carbon emissions with respect to SAT. (A,B) Annual mean active layer thickness (ALT), **(C,D)** annual mean total ecosystem carbon, and **(E,F)** annual CH_4 emission anomalies relative to the year 2000 as a function of SAT. All values are averaged over the initial permafrost domain **(A-C)** with high organic matter content (> 75 kgC/m^2; organic soils) and **(D-F)** with relatively low organic matter content (< 25 kgC/m^2; mineral soils) based on de Vrese & Brovkin, (2021) (*49*). All values are averaged over the initial permafrost domain and smoothed by the 11-year moving average.

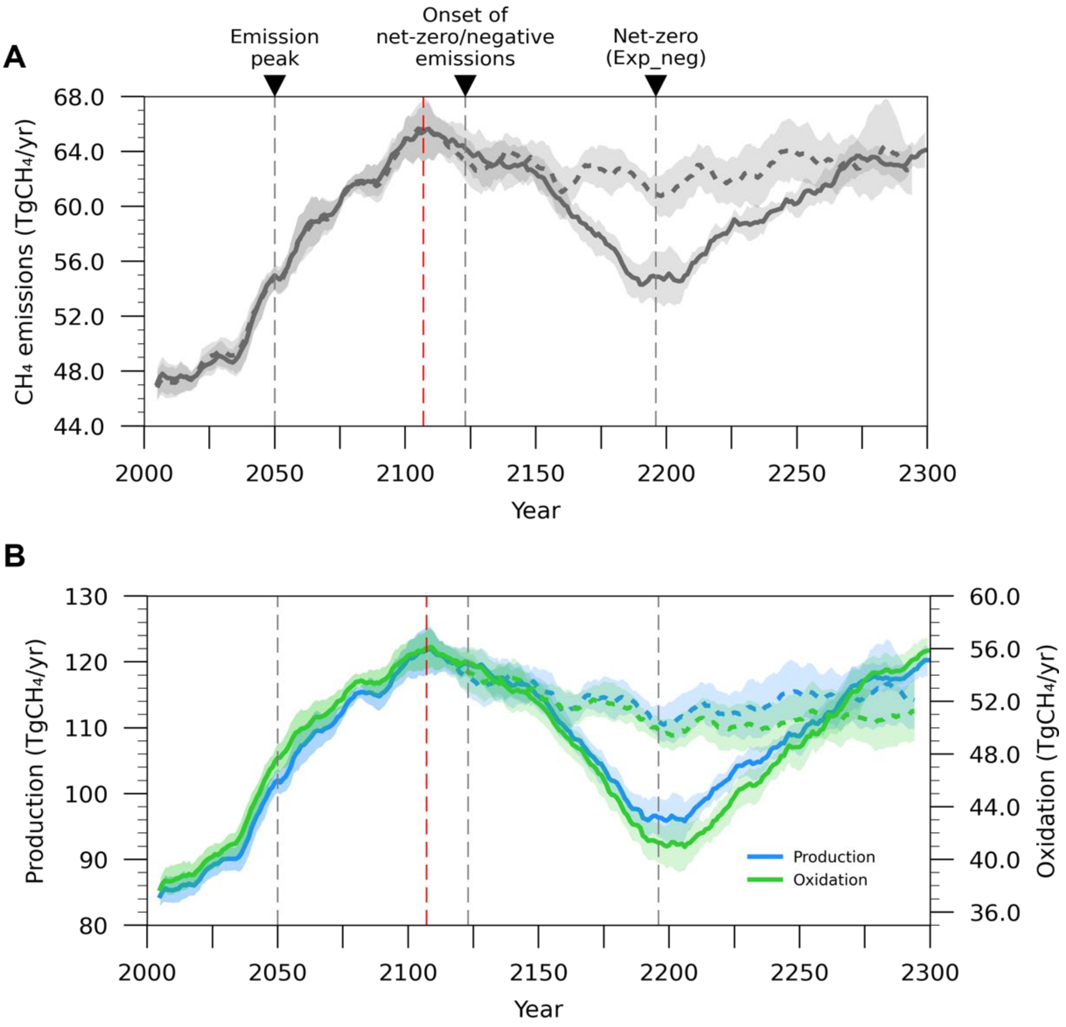

Fig. S4. Evolution of CH_4 emissions, production, and oxidation in permafrost region. (A) Time-series of the 11-year running mean of annual CH_4 emissions, **(B)** CH_4 production, and oxidation of CH_4 and CO_2. All values are averaged over the initial permafrost domain. The solid and dashed lines show the ensemble mean values for Exp_zero and Exp_neg, respectively. The shading indicates the 95% confidence interval based on the bootstrap method. The peak of anthropogenic emissions and the onset of negative emissions are indicated by the gray dashed vertical line. The point of maximum CO_2 levels is indicated by the red dashed vertical line.

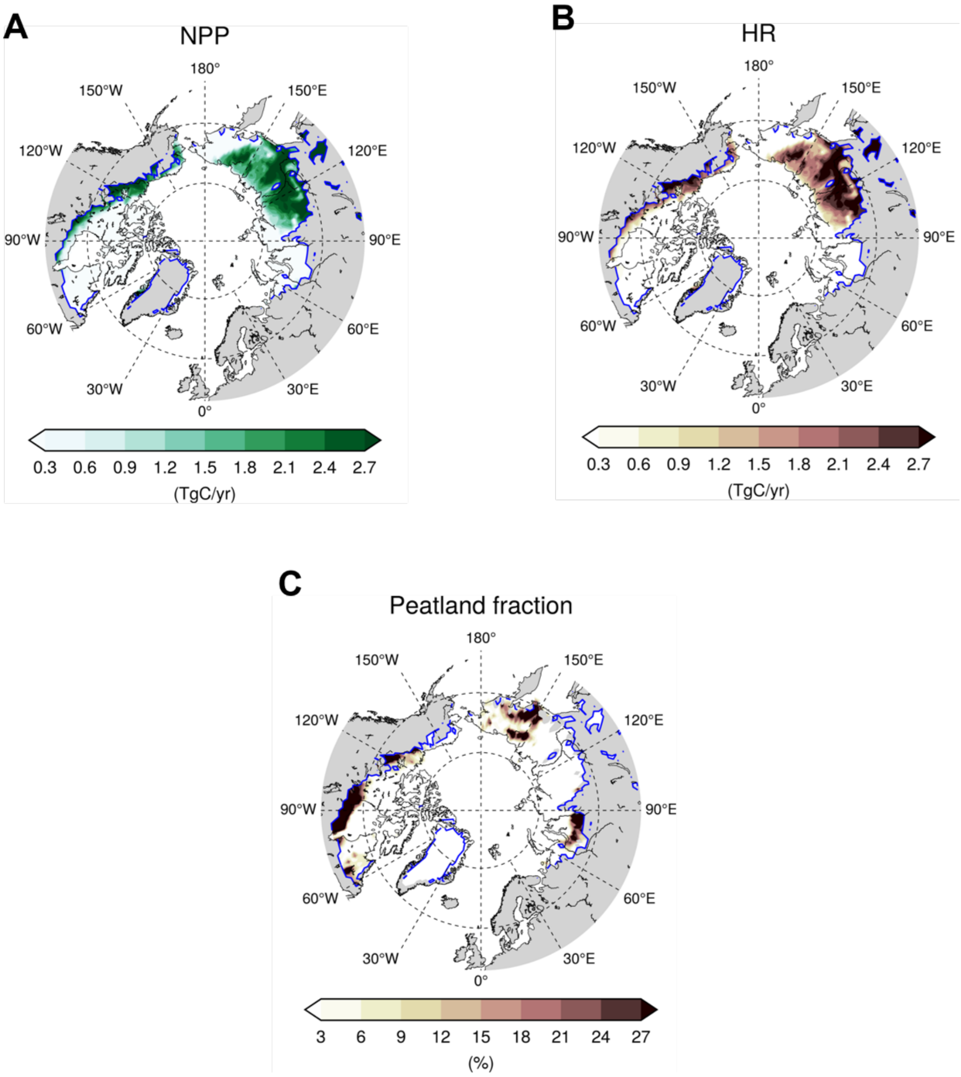

Fig. S5. Spatial pattern of NPP, HR, and peatland fraction in permafrost region. (A) Spatial map of initial NPP and **(B)** HR (year 2000) in the permafrost region. **(C)** Peatland fraction derived from three vector datasets: Olson et al. (2001) (*64*), Tarnocai et al. (2011) (*65*), and Lehner and Döll (2004) (*66*) (Details in Lawrence et al. 2018) (*43*). We note that the peatland fraction data are not used for processes related to the heterotrophic respiration and CH_4 production, but only for peatland fire.

REFERENCES AND NOTES

1. M. R. Allen, D. J. Frame, C. Huntingford, C. D. Jones, J. A. Lowe, M. Meinshausen, N. Meinshausen, Warming caused by cumulative carbon emissions towards the trillionth tonne. *Nature* **458**, 1163–1166 (2009).

2. T. A. Carleton, S. M. Hsiang, Social and economic impacts of climate. *Science* **353**, aad9837 (2016).

3. IPCC, Long-term climate change: Projections, commitments and irreversibility, in *Climate Change 2013: The Physical Science Basis. Contribution of Working Group I to the Fifth Assessment Report of the Intergovernmental Panel on Climate Change*, T. F. Stocker, D. Qin, G.-K. Plattner, M. Tignor, S. K. Allen, J. Boschung, A. Nauels, Y. Xia, V. Bex, P. M. Midgley, Eds. (Cambridge Univ. Press, 2013), pp. 1029–1136.

4. H. Matthews, N. Gillett, P. Stott, K. Zickfeld, The proportionality of global warming to cumulative carbon emissions. *Nature* **459**, 829–832 (2009).

5. United Nations Framework on Climate Change (UNFCCC), Adoption of the Paris Agreement, 21st Conference of the Parties (2015); http://unfccc.int/resource/docs/2015/cop21/eng/l09r01.pdf.

6. T. Gasser, C. Guivarch, K. Tachiiri, C. D. Jones, P. Ciais, Negative emissions physically needed to keep global warming below 2 °C. *Nat. Commun.* **6**, 7958 (2015).

7. E. Kriegler, G. Luderer, N. Bauer, L. Baumstark, S. Fujimori, A. Popp, J. Rogelj, J. Strefler, D. P. van Vuuren, Pathways limiting warming to 1.5°C: A tale of turning around in no time? *Philos. Trans. A Math. Phys. Eng. Sci.* **376**, 20160457 (2018).

8. J. Rogelj, M. Schaeffer, P. Friedlingstein, N. P. Gillett, D. P. Van Vuuren, K. Riahi, M. Allen, R. Knutti, Differences between carbon budget estimates unravelled. *Nat. Clim. Chang.* **6**, 245–252 (2016).

9. IPCC, 2018: Summary for Policymakers in *Global Warming of 1.5°C. An IPCC Special Report on the impacts of global warming of 1.5°C above pre-industrial levels and related*

global greenhouse gas emission pathways, in the context of strengthening the global response to the threat of climate change, sustainable development, and efforts to eradicate poverty, Masson-Delmotte, V., P. Zhai, H.-O. Pörtner, D. Roberts, J. Skea, P. R. Shukla, A. Pirani,W. Moufouma-Okia, C. Péan, R. Pidcock, S. Connors, J. B. R. Matthews, Y. Chen, X. Zhou,M. I. Gomis, E. Lonnoy, T. Maycock, M. Tignor, and T. Waterfield, Eds. (Cambridge Univ. Press, 2022), pp. 3–24; https://doi.org/10.1017/9781009157940.001.

10. J. Schwinger, A. Asaadi, N. J. Steinert, H. Lee, Emit now, mitigate later? Earth system reversibility under overshoots of different magnitudes and durations. *Earth Syst. Dynam.* **13**, 1641–1665 (2022).

11. O. Boucher, P. R. Halloran, E. J. Burke, M. Doutriaux-Boucher, C. D. Jones, J. Lowe, M. A. Ringer, E. Robertson, P. Wu, Reversibility in an Earth System model in response to CO_2 concentration changes. *Environ. Res. Lett.* **7**, 024013 (2012).

12. L. Cao, G. Bala, K. Caldeira, Why is there a short-term increase in global precipitation in response to diminished CO_2 forcing? *Geophys. Res. Lett.* **38**, L06703 (2011).

13. T. L. Frölicher, F. Joos, Reversible and irreversible impacts of greenhouse gas emissions in multi-century projections with the NCAR global coupled carbon cycle-climate model. *Climate Dynam.* **35**, 1439–1459 (2010).

14. A. Jeltsch-thömmes, T. F. Stocker, F. Joos, Hysteresis of the Earth system under positive and negative CO_2 emissions. *Environ. Res. Lett.* **15**, 124026 (2020).

15. P. Wu, J. Ridley, A. Pardaens, R. Levine, J. Lowe, The reversibility of CO_2 induced climate change. *Climate Dynam.* **45**, 745–754 (2015).

16. K. B. Tokarska, K. Zickfeld, The effectiveness of net negative carbon dioxide emissions in reversing anthropogenic climate change. *Environ. Res. Lett.* **10**, 094013 (2015).

17. A. H. MacDougall, Reversing climate warming by artificial atmospheric carbon-dioxide removal: Can a Holocene-like climate be restored? *Geophys. Res. Lett.* **40**, 5480–5485 (2013).

18. C. Voigt, M. E. Marushchak, B. W. Abbott, C. Biasi, B. Elberling, S. D. Siciliano, O. Sonnentag, K. J. Stewart, Y. Yang, P. J. Martikainen, Nitrous oxide emissions from permafrost-affected soils. *Nat. Rev. Earth Environ.* **1**, 420–434 (2020).

19. E. A. G. Schuur, B. W. Abbott, R. Commane, J. Ernakovich, E. Euskirchen, G. Hugelius, G. Grosse, M. Jones, C. Koven, V. Leshyk, D. Lawrence, M. M. Loranty, M. Mauritz, D. Olefeldt, S. Natali, H. Rodenhizer, V. Salmon, C. Schädel, J. Strauss, C. Treat, M. Turetsky, Permafrost and climate change: Carbon cycle feedbacks from the warming arctic. *Annu. Rev. Env. Resour.* **47**, 343–371 (2022).

20. A. H. MacDougall, K. Zickfeld, R. Knutti, H. D. Matthews, Sensitivity of carbon budgets to permafrost carbon feedbacks and non-CO_2 forcings. *Environ. Res. Lett.* **10**, 125003 (2015).

21. E. A. G. Schuur, J. Bockheim, J. G. Canadell, E. Euskirchen, C. B. Field, S. V. Goryachkin, S. Hagemann, P. Kuhry, P. M. Lafleur, H. Lee, G. Mazhitova, F. E. Nelson, A. Rinke, V. E. Romanovsky, N. Shiklomanov, C. Tarnocai, S. Venevsky, J. G. Vogel, S. A. Zimov, Vulnerability of permafrost carbon to climate change: Implications for the global carbon cycle. *Bioscience* **58**, 701–714 (2008).

22. E. A. G. Schuur, A. D. McGuire, C. Schädel, G. Grosse, J. W. Harden, D. J. Hayes, G. Hugelius, C. D. Koven, P. Kuhry, D. M. Lawrence, S. M. Natali, D. Olefeldt, V. E. Romanovsky, K. Schaefer, M. R. Turetsky, C. C. Treat, J. E. Vonk, Climate change and the permafrost carbon feedback. *Nature* **520**, 171–179 (2015).

23. E. J. Burke, A. Ekici, Y. Huang, S. E. Chadburn, C. Huntingford, P. Ciais, P. Friedlingstein, S. Peng, G. Krinner, Quantifying uncertainties of permafrost carbon–climate feedbacks. *Biogeosciences* **14**, 3051–3066 (2017).

24. J. A. Lowe, D. Bernie, The impact of Earth system feedbacks on carbon budgets and climate response. *Philos. Trans. R. Soc. A* **376**, 20170263 (2018).

25. K. Schaefer, H. Lantuit, V. E. Romanovsky, E. A. G. Schuur, R. Witt, The impact of the permafrost carbon feedback on global climate. *Environ. Res. Lett.* **9**, 085003 (2014).

26. K. R. Miner, M. R. Turetsky, E. Malina, A. Bartsch, J. Tamminen, A. D. McGuire, A. Fix, C. Sweeney, C. D. Elder, C. E. Miller, Permafrost carbon emissions in a changing Arctic. *Nat. Rev. Earth Environ.* **3**, 55–67 (2022).

27. Z. Zhu, S. Piao, R. B. Myneni, M. Huang, Z. Zeng, J. G. Canadell, P. Ciais, S. Sitch, P. Friedlingstein, A. Arneth, C. Cao, L. Cheng, E. Kato, C. Koven, Y. Li, X. Lian, Y. Liu, R. Liu, J. Mao, Y. Pan, S. Peng, J. Peñuelas, B. Poulter, T. A. M. Pugh, B. D. Stocker, N. Viovy, X. Wang, Y. Wang, Z. Xiao, H. Yang, S. Zaehle, N. Zeng, Greening of the Earth and its drivers. *Nat. Clim. Chang.* **6**, 791–795 (2016).

28. C. D. Koven, D. M. Lawrence, W. J. Riley, Permafrost carbon−climate feedback is sensitive to deep soil carbon decomposability but not deep soil nitrogen dynamics. *Proc. Natl. Acad. Sci. U.S.A.* **112**, 3752–3757 (2015).

29. S.-W. Park, J.-S. Kug, A decline in atmospheric CO_2 levels under negative emissions may enhance carbon retention in the terrestrial biosphere. *Commun. Earth Environ.* **3**, 289 (2022).

30. A. V. Eliseev, P. F. Demchenko, M. M. Arzhanov, I. I. Mokhov, Transient hysteresis of near-surface permafrost response to external forcing. *Climate Dynam.* **42**, 1203–1215 (2014).

31. C. D. Koven, V. K. Arora, P. Cadule, R. A. Fisher, C. D. Jones, D. M. Lawrence, J. Lewis, K. Lindsay, S. Mathesius, M. Meinshausen, M. Mills, Z. Nicholls, B. M. Sanderson, R. Séférian, N. C. Swart, W. R. Wieder, K. Zickfeld, Multi-century dynamics of the climate and carbon cycle under both high and net negative emissions scenarios. *Earth Syst. Dynam.* **13**, 885–909 (2022).

32. C. D. Jones, V. Arora, P. Friedlingstein, L. Bopp, V. Brovkin, J. Dunne, H. Graven, F. Hoffman, T. Ilyina, J. G. John, M. Jung, M. Kawamiya, C. Koven, J. Pongratz, T. Raddatz, J. T. Randerson, S. Zaehle, C4MIP–The Coupled Climate–Carbon Cycle Model Intercomparison Project: Experimental protocol for CMIP6. *Geosci. Model Dev.* **9**, 2853–2880 (2016).

33. C. D. Koven, B. M. Sanderson, A. L. S. Swann, Much of zero emissions commitment occurs before reaching net zero emissions. *Environ. Res. Lett.* **18**, 014017 (2023).

34. J. Schwinger, A. Asaadi, N. Goris, H. Lee, Possibility for strong northern hemisphere high-latitude cooling under negative emissions. *Nat. Commun.* **13**, 1095 (2022).

35. G. Danabasoglu, J.-F. Lamarque, J. Bacmeister, D. A. Bailey, A. K. DuVivier, J. Edwards, L. K. Emmons, J. Fasullo, R. Garcia, A. Gettelman, C. Hannay, M. M. Holland, W. G. Large, P. H. Lauritzen, D. M. Lawrence, J. T. M. Lenaerts, K. Lindsay, W. H. Lipscomb, M. J. Mills, R. Neale, K. W. Oleson, B. Otto-Bliesner, A. S. Phillips, W. Sacks, S. Tilmes, L. van Kampenhout, M. Vertenstein, A. Bertini, J. Dennis, C. Deser, C. Fischer, B. Fox-Kemper, J. E. Kay, D. Kinnison, P. J. Kushner, V. E. Larson, M. C. Long, S. Mickelson, J. K. Moore, E. Nienhouse, L. Polvani, P. J. Rasch, W. G. Strand, The Community Earth System Model Version 2 (CESM2). *J. Adv. Model. Earth Syst.* **12**, e2019MS001916 (2020).

36. N. Steinert, M. Debolskiy, E. Burke, F. García-Pereira, H. Lee, Evaluating permafrost definitions for global permafrost area estimates in CMIP6 climate models. *Environ. Res. Lett.* **19**, 014033 (2023).

37. S.-I. An, J. Shin, S.-W. Yeh, S.-W. Son, J.-S. Kug, S.-K. Min, H.-J. Kim, Global cooling hiatus driven by an AMOC overshoot in a carbon dioxide removal scenario. *Earths Future 9*, e2021EF002165 (2021).

38. J.-S. Kug, J.-H. Oh, S.-I. An, S.-W. Yeh, S.-K. Min, S.-W. Son, J. Kam, Y.-G. Ham, J. Shin, Hysteresis of the intertropical convergence zone to CO_2 forcing. *Nat. Clim. Chang.* **12**, 47–53 (2022).

39. H. Lee, A. Ekici, J. Tjiputra, H. Muri, S. E. Chadburn, D. M. Lawrence, J. Schwinger, The response of permafrost and high-latitude ecosystems under large-scale stratospheric aerosol injection and its termination. *Earths Future* **7**, 605–614 (2019).

40. G. B. Bonan, *Climate Change and Terrestrial Ecosystem Modeling* (Cambridge Univ. Press, 2019).

41. S.-W. Park, K. M. Noh, S.-I. An, J. Kam, E.-Y. Kwon, S.-K. Min, R. Park, S.-W. Son, S.-W. Yeh,J.-S. Kug, How will global carbon cycle respond to negative emissions? ESS Open Archive (2023).

42. W. J. Riley, Z. M. Subin, D. M. Lawrence, S. C. Swenson, M. S. Torn, L. Meng, N. M. Mahowald, P. Hess, Barriers to predicting changes in global terrestrial methane fluxes: Analyses using CLM4Me, a methane biogeochemistry model integrated in CESM. *Biogeosciences.* **8**, 1925–1953 (2011).

43. D. Lawrence, R. Fisher, C. Koven, K. Oleson, S. Swenson, M. Vertenstein, B. Andre, G. Bonan, B. Ghimire, L. van Kampenhout, D. Kennedy, E. Kluzek, R. Knox, P. Lawrence, F. Li, H. Li,D. Lombardozzi, Y. Lu, J. Perket, W. Riley, W. Sacks, M. Shi, W. Wieder, C. Xu, A. Ali,A. Badger, G. Bisht, P. Broxton, M. Brunke, J. Buzan, M. Clark, T. Craig, K. Dahlin, B. Drewniak, L. Emmons, J. Fisher, M. Flanner, P. Gentine, J. Lenaerts, S. Levis, Technical Description of version 5.0 of the Community Land Model (CLM) [National Center for Atmospheric Research (NCAR), 2018].

44. D. M. Lawrence, C. D. Koven, S. C. Swenson, W. J. Riley, A. G. Slater, Permafrost thaw and resulting soil moisture changes regulate projected high-latitude CO_2 and CH_4 emissions. *Environ. Res. Lett.* **10**, 94011 (2015).

45. D. Olefeldt, M. R. Turetsky, P. M. Crill, A. D. McGuire, Environmental and physical controls on northern terrestrial methane emissions across permafrost zones. *Glob. Chang. Biol.* **19**, 589–603 (2013).

46. H. Lee, S. C. Swenson, A. G. Slater, D. M. Lawrence, Effects of excess ground ice on projections of permafrost in a warming climate. *Environ. Res. Lett.* **9**, 124006 (2014).

47. C. C. Treat, S. M. Natali, J. Ernakovich, C. M. Iversen, M. Lupascu, A. D. McGuire, R. J. Norby, T. Roy Chowdhury, A. Richter, H. Šantrůčková, C. Schädel, E. A. G. Schuur, V. L. Sloan, M. R. Turetsky, M. P. Waldrop, A pan-Arctic synthesis of CH_4 and CO_2 production from anoxic soil incubations. *Glob. Chang. Biol.* **21**, 2787–2803 (2015).

48. J. Huang, B. Mendoza, J. S. Daniel, C. J. Nielsen, L. Rotstayn, O. Wild, Anthropogenic and natural radiative forcing, *Clim. Chang. 2013 Phys. Sci. Basis Work. Gr. I Contrib. to Fifth Assess. Rep. Intergov. Panel Clim. Chang.* (Cambridge Univ. Press, 2013), pp. 659–740, 9781107057999.

49. P. de Vrese, V. Brovkin, Timescales of the permafrost carbon cycle and legacy effects of temperature overshoot scenarios. *Nat. Commun.* **12**, 2688 (2021).

50. S.-K. Kim, J. Shin, S.-I. An, H.-J. Kim, N. Im, S.-P. Xie, J.-S. Kug, S.-W. Yeh, Widespread irreversible changes in surface temperature and precipitation in response to CO_2 forcing. *Nat. Clim. Chang.* **12**, 834–840 (2022).

51. G. Hugelius, J. Strauss, S. Zubrzycki, J. W. Harden, E. A. G. Schuur, C.-L. Ping, L. Schirrmeister, G. Grosse, G. J. Michaelson, C. D. Koven, J. A. O'Donnell, B. Elberling, U. Mishra, P. Camill, Z. Yu, J. Palmtag, P. Kuhry, Estimated stocks of circumpolar permafrost carbon with quantified uncertainty ranges and identified data gaps. *Biogeosciences* **11**, 6573–6593 (2014).

52. G. Hugelius, J. Loisel, S. Chadburn, R. B. Jackson, M. Jones, G. MacDonald, M. Marushchak, D. Olefeldt, M. Packalen, M. B. Siewert, C. Treat, M. Turetsky, C. Voigt, Z. Yu, Large stocks of peatland carbon and nitrogen are vulnerable to permafrost thaw. *Proc. Natl. Acad. Sci. U.S.A.* **117**, 20438–20446 (2020).

53. E. J. Burke, Y. Zhang, G. Krinner, Evaluating permafrost physics in the Coupled Model Intercomparison Project 6 (CMIP6) models and their sensitivity to climate change. *Cryosphere* **14**, 3155–3174 (2020).

54. A. Ekici, H. Lee, D. M. Lawrence, S. C. Swenson, C. Prigent, Ground subsidence effects on simulating dynamic high-latitude surface inundation under permafrost thaw using CLM5. *Geosci. Model Dev.* **12**, 5291–5300 (2019).

55. M. R. Turetsky, B. W. Abbott, M. C. Jones, K. W. Anthony, D. Olefeldt, E. A. G. Schuur, G. Grosse, P. Kuhry, G. Hugelius, C. Koven, D. M. Lawrence, C. Gibson, A. B. K. Sannel, A. D. McGuire, Carbon release through abrupt permafrost thaw. *Nat. Geosci.* **13**, 138–143 (2020).

56. J. Nitzbon, T. Schneider von Deimling, M. Aliyeva, S. Chadburn, G. Grosse, S. Laboor, H. Lee, G. Lohmann, N. Steinert, S. Stuenzi, M. Werner, S. Westermann, M. Langer, No respite from permafrost-thaw impacts in absence of a global tipping point. *Nat. Clim. Chang.* **14**, 573–585 (2023).

57. C. M. Gibson, L. E. Chasmer, D. K. Thompson, W. L. Quinton, M. D. Flannigan, D. Olefeldt, Wildfire as a major driver of recent permafrost thaw in boreal peatlands. *Nat. Commun.* **9**, 3041 (2018).

58. K. Van Huissteden, *Thawing Permafrost: Permafrost Carbon in a Warming Arctic* (Springer, 2020).

59. J. B. Fisher, M. Sikka, W. C. Oechel, D. N. Huntzinger, J. R. Melton, C. D. Koven, A. Ahlström, M. A. Arain, I. Baker, J. M. Chen, P. Ciais, C. Davidson, M. Dietze, B. El-Masri, D. Hayes, C. Huntingford, A. K. Jain, P. E. Levy, M. R. Lomas, B. Poulter, D. Price, A. K. Sahoo, K. Schaefer, H. Tian, E. Tomelleri, H. Verbeeck, N. Viovy, R. Wania, N. Zeng, C. E. Miller, Carbon cycle uncertainty in the Alaskan Arctic. *Biogeosciences* **11**, 4271–4288 (2014).

60. D. M. Lawrence, R. A. Fisher, C. D. Koven, K. W. Oleson, S. C. Swenson, G. Bonan, N. Collier, B. Ghimire, L. van Kampenhout, D. Kennedy, E. Kluzek, P. J. Lawrence, F. Li, H. Li, D. Lombardozzi, W. J. Riley, W. J. Sacks, M. Shi, M. Vertenstein, W. R. Wieder, C. Xu, A. A. Ali, A. M. Badger, G. Bisht, M. van den Broeke, M. A. Brunke, S. P. Burns, J. Buzan, M. Clark, A. Craig, K. Dahlin, B. Drewniak, J. B. Fisher, M. Flanner, A. M. Fox, P. Gentine, F. Hoffman, G. Keppel-Aleks, R. Knox, S. Kumar, J. Lenaerts, L. R. Leung, W. H. Lipscomb, Y. Lu, A. Pandey, J. D. Pelletier, J. Perket, J. T. Randerson, D. M. Ricciuto, B. M. Sanderson, A. Slater, Z. M. Subin, J. Tang, R. Q. Thomas, M. Val Martin, X. Zeng, The Community Land Model Version 5: Description of new features, benchmarking, and impact of forcing uncertainty. *J. Adv. Model Earth Syst.* **11**, 4245–4287 (2019).

61. C. Koven, P. Friedlingstein, P. Ciais, D. Khvorostyanov, G. Krinner, C. Tarnocai, On the formation of high-latitude soil carbon stocks: Effects of cryoturbation and insulation by organic matter in a land surface model. *Geophys. Res. Lett.* **36**, L21501 (2009).

62. J. G. Bockheim, I. B. Campbell, M. McLeod, Permafrost distribution and active-layer depths in the McMurdo Dry Valleys,Antarctica. *Permafr. Periglac. Process.* **18**, 217–227 (2007).

63. A. H. MacDougall, T. L. Frölicher, C. D. Jones, J. Rogelj, H. D. Matthews, K. Zickfeld, V. K. Arora, N. J. Barrett, V. Brovkin, F. A. Burger, M. Eby, A. V. Eliseev, T. Hajima, P. B. Holden,

A. Jeltsch-Thömmes, C. Koven, N. Mengis, L. Menviel, M. Michou, I. I. Mokhov, A. Oka, J. Schwinger, R. Séférian, G. Shaffer, A. Sokolov, K. Tachiiri, J. Tjiputra, A. Wiltshire, T. Ziehn, Is there warming in the pipeline? A multi-model analysis of the Zero Emissions Commitment from CO_2. *Biogeosciences* **17**, 2987–3016 (2020).

64. D. M. Olson, E. Dinerstein, E. D. Wikramanayake, N. D. Burgess, G. V. N. Powell, E. C. Underwood, J. A. D'amico, I. Itoua, H. E. Strand, J. C. Morrison, C. J. Loucks, T. F. Allnutt, T. H. Ricketts, Y. Kura, J. F. Lamoreux, W. W. Wettengel, P. Hedao, K. R. Kassem, Terrestrial ecoregions of the world: A new map of life on Earth: A new global map of terrestrial ecoregions provides an innovative tool for conserving biodiversity. *Bioscience* **51**, 933–938 (2001).

65. C. Tarnocai, I. M. Kettles, B. Lacelle, Peatlands of Canada, Geological Survey of Canada, Open File 6561, CD-ROM. (2011).

66. B. Lehner, P. Döll, Development and validation of a global database of lakes, reservoirs and wetlands. *J. Hydrol.* **296**, 1–22 (2004).
